# Production of a functional cell wall-anchored minicellulosome by recombinant *Clostridium acetobutylicum* ATCC 824

**DOI:** 10.1186/s13068-016-0526-x

**Published:** 2016-05-23

**Authors:** Benjamin J. Willson, Katalin Kovács, Tom Wilding-Steele, Robert Markus, Klaus Winzer, Nigel P. Minton

**Affiliations:** Clostridia Research Group, BBSRC/EPSRC Synthetic Biology Research Centre, School of Life Sciences, University of Nottingham, Nottingham, NG7 2RD UK; SLIM Imaging Unit, Faculty of Medicine and Health Sciences, School of Life Sciences, University of Nottingham, Nottingham, NG7 2RD UK

**Keywords:** Cellulosome, Scaffoldin, Sortase anchoring, Consolidated bioprocessing, *Clostridium acetobutylicum*

## Abstract

**Background:**

The use of fossil fuels is no longer tenable. Not only are they a finite resource, their use is damaging the environment through pollution and global warming. Alternative, environmentally friendly, renewable sources of chemicals and fuels are required. To date, the focus has been on using lignocellulose as a feedstock for microbial fermentation. However, its recalcitrance to deconstruction is making the development of economic processes extremely challenging. One solution is the generation of an organism suitable for use in consolidated bioprocessing (CBP), i.e. one able to both hydrolyse lignocellulose and ferment the released sugars, and this represents an important goal for synthetic biology. We aim to use synthetic biology to develop the solventogenic bacterium *C. acetobutylicum* as a CBP organism through the introduction of a cellulosome, a complex of cellulolytic enzymes bound to a scaffold protein called a scaffoldin. In previous work, we were able to demonstrate the in vivo production of a *C. thermocellum*-derived minicellulosome by recombinant strains of *C. acetobutylicum*, and aim to develop on this success, addressing potential issues with the previous strategy.

**Results:**

The genes for the cellulosomal enzymes Cel9G, Cel48F, and Xyn10A from *C. cellulolyticum* were integrated into the *C. acetobutylicum* genome using Allele-Coupled Exchange (ACE) technology, along with a miniscaffoldin derived from *C. cellulolyticum* CipC. The possibility of anchoring the recombinant cellulosome to the cell surface using the native sortase system was assessed, and the cellulolytic properties of the recombinant strains were assayed via plate growth, batch fermentation and sugar release assays.

**Conclusions:**

We have been able to demonstrate the synthesis and in vivo assembly of a four-component minicellulosome by recombinant *C. acetobutylicum* strains. Furthermore, we have been able to anchor a minicellulosome to the *C. acetobutylicum* cell wall by the use of the native sortase system. The recombinant strains display an improved growth phenotype on xylan and an increase in released reducing sugar from several substrates including untreated powdered wheat straw. This constitutes an important milestone towards the development of a truly cellulolytic strain suitable for CBP.

**Electronic supplementary material:**

The online version of this article (doi:10.1186/s13068-016-0526-x) contains supplementary material, which is available to authorized users.

## Background

In recent years, biofuels such as bioethanol have received increasing attention as potential alternatives to fossil fuels. However, the use of ‘first generation’ biofuels, i.e. those produced using food crop-derived sugars as feedstock, is not without its issues. The use of food stocks may lead to elevated prices as a result of increased demand, while the use of limited water resources and agricultural land will reduce the land available for food production [1, 2]. More attractive would be to use lignocellulosic biomass, a complex material which makes up the cell walls of plants [3]. Lignocellulose consists primarily of cellulose, a β1, 4-linked polymer of glucose; other components are hemicelluloses, branched polymers of xylose, glucose, and/or mannose [4], and lignin, a polymer of aromatic alcohols [5]. As a feedstock for microbial fermentations, the most readily available sources of biomass include agricultural and domestic wastes as well as dedicated crops such as willow and switchgrass [6]. However, lignocellulose is notoriously recalcitrant to deconstruction. Its exploitation is reliant on an energy intensive pre-treatment step [7] and, thereafter, the addition of costly exogenous hydrolytic enzymes required to convert the partially deconstructed biomass into the sugars needed by the fermentative process organisms. The costs involved are making the development of economic processes extremely challenging.

To improve process economics, the concept of consolidated bioprocessing (CBP) has been proposed, where a single organism or consortium is able to both degrade lignocellulose and ferment all the released sugars [8]. As no suitable organism has been isolated from nature, the generation of a CBP organism presents a challenging goal for synthetic biology.

At present, efforts to develop an organism for CBP fall into two categories [8]. In the ‘native’ strategy, an organism that is able to efficiently degrade lignocellulose is engineered for biofuel production. This can involve the improvement of the characteristics or yield of a native solvent producer such as *Clostridium thermocellum* [9] (*Ruminiclostridium thermocellum* [10]) or the introduction of new metabolic pathways into a non-solventogenic organism such as *Clostridium cellulovorans* [11]. Conversely, the ‘recombinant’ strategy involves the engineering of well-established solvent producers to use lignocellulose as a feedstock through the expression of cellulolytic enzymes. Several studies so far have focused on the ethanol-producing yeast *Saccharomyces cerevisiae* [12, 13]. Nevertheless, the recent retraction of an article purporting to show the development of a strain of *Bacillus subtilis* able to grow on lignocellulosic biomass highlights the challenges of engineering organisms for CBP [14].

One organism that is of particular interest as a potential CBP chassis is *Clostridium acetobutylicum,* a Gram-positive, spore forming, obligate anaerobe that belongs to the group I clostridia [15, 16]. *C. acetobutylicum* is able to rapidly convert sugars into solvents through the acetone–butanol–ethanol (ABE) fermentation pathway. It was formerly used from the First World War onwards on an industrial scale, initially to produce acetone and subsequently for the production of butanol. By the middle of the last century, however, the price of feedstocks made the ABE process uneconomic compared to the production of butanol by the petrochemical industry, resulting in the closure of ABE plants [17]. In recent times, interest in butanol has intensified due to its superior properties as a biofuel compared to ethanol [18]. This has provided the impetus to reappraise the benefits of the ABE process. *C. acetobutylicum*, while unable to natively utilise lignocellulose, is able to utilise all the hexose and pentose sugars that result from its deconstruction [19]. By engineering cellulolytic activity into *C. acetobutylicum*, it may be possible to create a strain that is capable of fermenting lignocellulosic material, making butanol production through ABE fermentation both sustainable and cost effective.

In nature, lignocellulose is broken down by specialised organisms using one of two strategies [20]. The first involves the secretion of a wide variety of free cellulolytic enzymes into the environment, whereas the second is reliant on the production of a large extracellular complex called a cellulosome. In cellulosomal systems, enzymes are anchored to a scaffolding protein called a ‘scaffoldin’ as a result of interactions between the cohesin domains of the scaffoldin and the dockerin domains of the enzymes. In most cases, the incorporation into the scaffoldin of a carbohydrate binding module (CBM) with specificity for cellulose allows the complex to adhere to the substrate [21]. Cellulosomal systems are generally found in anaerobic bacteria and are hypothesised to be an adaptation to the limitations of an anaerobic environment; the assembly of the enzymes into a complex allows greater synergy between enzymes with different activities, potentially reduces the competition for binding sites on the substrate, and allows the anchoring of the complex to the cell [22, 23]. Interestingly, *C. acetobutylicum* is able to produce a cellulosome [24], although it appears to be inactive.

The potential of *C. acetobutylicum* for CBP has not gone unnoticed and, to date, a number of studies have focused on the heterologous expression of cellulases and cellulosomal components with this organism. However, expression of many of these components has proven difficult. While miniscaffoldins [25, 26] and several cellulosomal enzymes [26–28] have been successfully overexpressed, certain enzymes, such as Cel9G, Cel48F and Cel9E from *Clostridium cellulolyticum* (*Ruminiclostridium cellulolyticum* [10]), have been shown to be detrimental when their genes are overexpressed on multicopy plasmid vectors. Nevertheless, in our previous work [29], we were able to successfully demonstrate the production of a number of cellulosomal enzymes, including Cel48S, a GH48 cellulase, from encoding genes that had been integrated into the *C. acetobutylicum* genome. While genomic integration of the genes appeared to resolve the toxicity issues associated with plasmid-based overexpression, the resulting strains were still unable to utilise lignocellulosic material. In this work, we aimed to address the potential issues that we had identified in our previous strategy, continuing the development of *C. acetobutylicum* as a CBP organism.

## Results and discussion

### Selection of cellulosomal components

Our previous work focused on the expression of cellulosomal genes sourced from a thermophile, *C. thermocellum*. Here, we chose to express cellulases from a mesophile, namely, *C. cellulolyticum*, as we suspected that enzymes from a mesophilic background may have a greater activity in cultures of *C. acetobutylicum*. We selected two glucanase enzymes for inclusion in our cellulosome constructs: Cel9G and Cel48F. Cel48F is a processive endocellulase [30] that is mainly active towards crystalline cellulose and is the most prevalent enzyme in *C. cellulolyticum* cellulosomes [31], whereas Cel9G is an endoglucanase [32], a major enzyme in *C. cellulolyticum* cellulosomes [31], and is mainly active towards soluble substrates such as CMC and glucan [33]. When in a complex with a cellulase such as Cel48F or Cel9E, Cel9G displays a very strong synergy against insoluble celluloses such as Avicel and bacterial cellulose [34]. Although Cel9G is not the most active *C. cellulolyticum* GH9 enzyme, the combination of Cel48F and Cel9G provides a higher rate of Avicel degradation than any other Cel48F/GH9 combination [33]. One possible explanation for this synergy is the action of the Cel9G carbohydrate binding module, CBM3c. While this domain does not bind as strongly to cellulose as the CBM3a and 3b domains of cellulosomal scaffoldins [35], it may disrupt the structure of the cellulose, providing more opportunity for enzymatic attack [36].

To further improve the activity of the cellulosomes towards ‘natural’ substrates, we also chose to express a xylanase. The removal of xylan has been shown to be necessary for the efficient breakdown of lignocellulose by synthetic minicellulosomes [37]; while *C. acetobutylicum* ATCC 824 does express xylanases [38], it is only capable of efficient growth on xylan as a sole carbon source when in a pH-controlled chemostat culture [39]. *C. acetobutylicum* is reportedly unable to grow on solid medium with xylan as a sole carbon source, and could only grow in batch culture if xylose was added to promote xylan metabolism [39]. We hypothesised that the native xylanases may not be efficiently expressed under the conditions that would be found in a potential fermentation of lignocellulose, and activity against lignocellulose may be more likely if a cellulosomal xylanase is constitutively expressed. For this purpose, we chose Xyn10A (Ccel_0931), a smaller enzyme consisting of only a GH10 domain and dockerin [40]. Xyn10A has been identified in *C. cellulolyticum* cellulosomes [31], albeit at a lesser level than Cel9G or Cel48F; while no direct experimental data are available for the activity of Xyn10A, it bears a high degree of homology to known endoxylanases such as XynB from *Thermotoga maritima* [40]. Interestingly, unlike other GH10 enzymes from *C. cellulolyticum*, Xyn10A appears to be upregulated when cells are grown in the presence of crystalline cellulose [41].

Previously [29], we expressed heterologous genes from synthetic operons integrated into the chromosome using Allele-Coupled Exchange (ACE) technology, with expression driven by the genomic P_*thl*_ and additional P_BB2*thl*OID_ promoters. However, we observed that in the longer operon constructs, the expression levels of the downstream components were significantly reduced. To address this issue, we decided to express our components from individual promoters. At present, ACE has been exemplified at two different loci in *C. acetobutylicum* [42]: the *pyrE* locus and the *thl* locus. Genes integrated at the *thl* locus are under the transcriptional control of the native thiolase gene (*thl*) promoter, P_*thl*_, whereas those integrated at the *pyrE* locus require the use of an additional promoter. In preliminary work, we were able to observe the in vivo formation of minicellulosomes (data not shown) from *C. acetobutylicum* strains with a mini-CipC scaffoldin integrated at the *thl* locus and with either Cel9G or Cel48F integrated at the *pyrE* locus under the control of the P_*fac*OID_ promoter (comprising the promoter of the *Clostridium pasteurianum* ferredoxin gene into which was inserted a single ideal *lac* operator). Thus, we chose to expand upon this strategy by integrating the scaffoldin-encoding gene at the *thl* locus and a gene cassette encoding hydrolase enzymes at the *pyrE* locus.

### Assembly and expression of heterologous cellulosome components

**Fig. 1:**
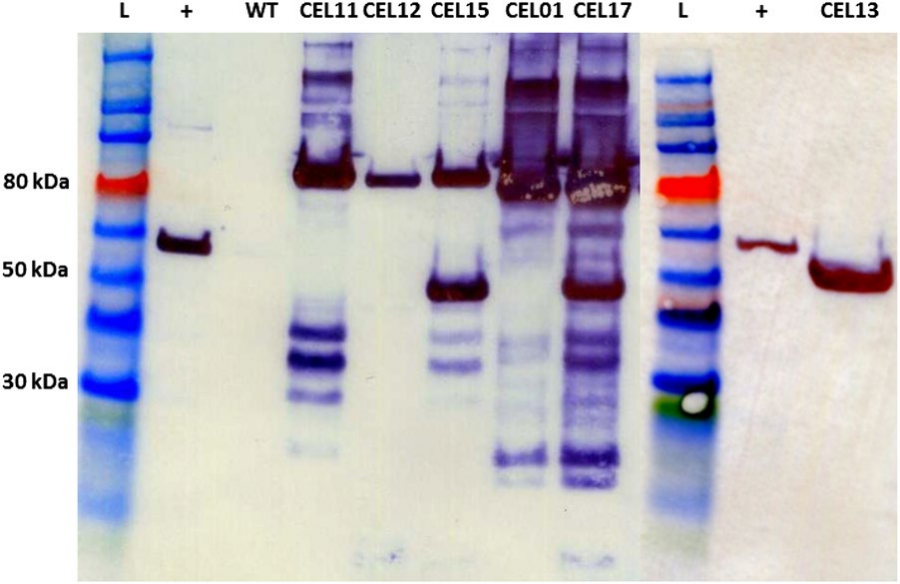
SDS-PAGE/western blot analysis of supernatants from recombinant *C. acetobutylicum* strains producing heterologous cellulosome components encoded by chromosomally integrated gene constructs. Supernatants were obtained at an OD_600_ of approximately 0.8 and proteins concentrated 100-fold by TCA precipitation before being separated on 4–12 % Bis–Tris gradient gels. All cellulosomal components have been engineered to bear the FLAG epitope tag at the C-terminus, allowing visualisation using ANTI-FLAG M2 monoclonal antibody-horseradish peroxidise conjugate. Supernatants were obtained from wild-type *C. acetobutylicum* (WT) and from strains CEL11, expressing a gene encoding Cel9G-FLAG, CEL12, expressing a gene encoding Cel48F-FLAG, CEL13, expressing a gene encoding Xyn10A-FLAG, CEL15, expressing genes encoding all three enzymes (Xyn10A-FLAG, Cel9G-FLAG, and Cel48F-FLAG), CEL01, expressing a gene encoding the BBCipC3-FLAG miniscaffoldin, and CEL17, expressing genes encoding all three enzymes and the BBCipC3-FLAG miniscaffoldin. L, ColorPlus Prestained Protein Ladder (10–230 kDa); +, carboxy terminal FLAG-BAP control protein (50 kDa)

A mini-CipC3 scaffoldin gene cassette, encoding the CBM, X domain, and first three cohesin domains of *C. cellulolyticum* CipC, was assembled from separate BB2 fragments and integrated at the *thl* locus through ACE in the same manner as the mini-CipA scaffoldins in our previous work [29]. In preliminary experiments, production of this construct was compared to a scarless variant synthesised as a single BB2 fragment, and no difference was observed (data not shown). Thus, we decided to continue with the BB2-assembled variant. However, integration of the enzyme-encoding gene modules was more complicated. To prevent homologous recombination within our enzyme-encoding cassette, it was necessary to use a unique promoter for each gene module. Two promoters were already available: the P_BB2*thl*OID_ promoter (the BB2-format *C. acetobutylicum* P_*thl*_ promoter containing a single ideal *lac* operator) used in our previous work [29], and the P_*fac*OID_ promoter used in preliminary experiments. A BB2-format variant of the P_*fac*Oid_ promoter, P_BB2*fac*Oid_, was generated following the same principles as the P_BB2*thl*Oid_ promoter, incorporating the RBS from the *C. acetobutylicum* P_*thl*_ promoter. A third promoter, P-_BB2*fdx*OID_, was designed, consisting of the promoter of the *C. sporogenes* ferredoxin gene (*fdx*) that incorporated a single ideal *lac* operator and P_*thl*_ RBS. Of these three promoters, the P_BB2*fac*OID_ promoter was found to be the strongest, whereas the P_BB2*thl*OID_ promoter was the weakest (data not shown). When the enzymes were expressed individually from the P_*fac*Oid_ promoter, we observed the Cel48F gene to exhibit the lowest expression level (Fig. 1). Thus, it was assigned the P_BB2*fac*OID_ promoter, in order to maximise the production of this important enzyme. As the Xyn10A gene had the highest level of expression, and was assumed to be of less overall importance, it was assigned the weaker P_BB2*thl*OID_ promoter. Thus, the Cel9G gene was assigned the intermediate-strength P_BB2*fdx*OID_ promoter.

To prevent possible interference between the promoters, we chose to place terminators between each gene. Due to the limited range of proven terminators in *C. acetobutylicum*, it was necessary to analyse a range of Rho-independent terminators. The *E. coli rrnB* T1 terminator and *B. subtilisgyrA* terminator were synthesised based on the existing sequences in the registry of standard biological parts [43] (BBa_B0010 and BBa_K780000, respectively). Additional terminators were synthesised based on the terminators of the *Lactococcus lactis pepN* gene [44], the *Lactobacillus acidophilus* and *Clostridium difficile* (*Peptoclostridium difficile* [10]) *slpA* genes [45, 46], the *B. subtilis* Φ29 phage late TD1 transcript [47], and the *B. subtilistyrS* tRNA gene [48]. The *E. colirrnB* T1 terminator, Φ29 TD1 terminator, *B. subtilistyrS* terminator, and *B. subtilis gyrA* terminator had all been characterised in other organisms and found to give high levels of efficiency [47–50], whereas the *L. lactis pepN* and *L. acidophilus slpA* terminators had some evidence of activity [51, 52]. After our own analysis of the efficacies of the terminators in *C. acetobutylicum* (Additional file 1: Figure S1), we selected the *B. subtilistyrS* tRNA terminator (TtyrS) and *E. coli rrnB* T1 terminator (EcoT1) for subsequent use. The gene cassette was finally assembled in *C. acetobutylicum* with the organisational encoding order of P_BB2*thl*Oid__Xyn10A-TtyrS-P_BB2*fdx*Oid__Cel9G-EcoT1-P_BB2*fac*Oid__Cel48F. Thus, the weakest promoter was placed before the first gene of the cassette, and the strongest at the end, so as to further minimise the effects of promoter interference.

While the use of *lac*-repressible promoters enabled the assembly of single- or double-gene constructs in *E. coli*, it was not possible to assemble the triple-gene cassette as a single unit, as expression of Cel48F and Cel9G had a toxic effect. This is likely to result from an inability to secrete the proteins efficiently, potentially due to blockage of the secretion system; overexpression of Cel48F has been observed to result in accumulations of Cel48F as inclusion bodies in the cytoplasm and periplasm, of which around 50 % had been processed by the secretion machinery but not exported [53]. Cel9G is also known to form inclusion bodies when produced in *E. coli* and may be subject to the same secretion defect [32].Plasmids carrying double-gene constructs were only successfully isolated after growth at 30 °C for 3 days on solid medium and for 48 h in liquid culture. Nevertheless, we were able to integrate the entire three-enzyme-encoding gene cassette into the genome in two consecutive steps through the use of iterative ACE [42]. The correct identity of the integrants was confirmed by nucleotide sequencing of appropriately amplified PCR products. The production of all three enzymes that would result from the integration of all three genes did not appear to have any significant negative impact on the growth phenotype of the strain.

Denaturing western blot analysis of the recombinant strains (Fig. 1) demonstrated that all the introduced cellulosome components can be produced and secreted by *C. acetobutylicum*. Although we have previously used the ACE technology to engineer *C. acetobutylicum* for production of cellulase enzymes [29], the enzymes chosen here have been previously established as being especially difficult to produce in *C. acetobutylicum* [27]. Thus, the expression of these enzymes provides a further exemplification of the utility of ACE technology for creating stable genomic insertions. Although Cel9G, Cel48F and CipC3 migrate at roughly the same speed, it is possible in the three-enzyme coexpression (XGF and XGF:C) samples to observe degradation products corresponding to each of the individual components: Cel9G produces degradation products migrating at roughly 35 and 40 kDa, Cel48F produces a roughly 5 kDa degradation product, and CipC3 produces a 15 kDa degradation product. It is important to note that expression levels of the components are not dependent solely on the strength of the promoter; when expressed from the P_*fac*OID_/P_BB2*fac*Oid_ promoter, the levels of Cel9G and Xyn10A produced are far greater than those of Cel48F. Using the weaker P_BB2*fdx*OID_ promoter for Cel9G, we reduced the level of Cel9G in the coexpression strain to more closely correspond to the level of Cel48F. However, the levels of Xyn10A produced in the coexpression strains are roughly equivalent to the levels of Cel9G and Cel48F combined, despite the use of the weaker P_BB2*thl*Oid_ promoter; when expressed alone from the P_BB2*fac*Oid_ promoter, the levels of Xyn10A appear to be even greater.

**Fig. 2:**
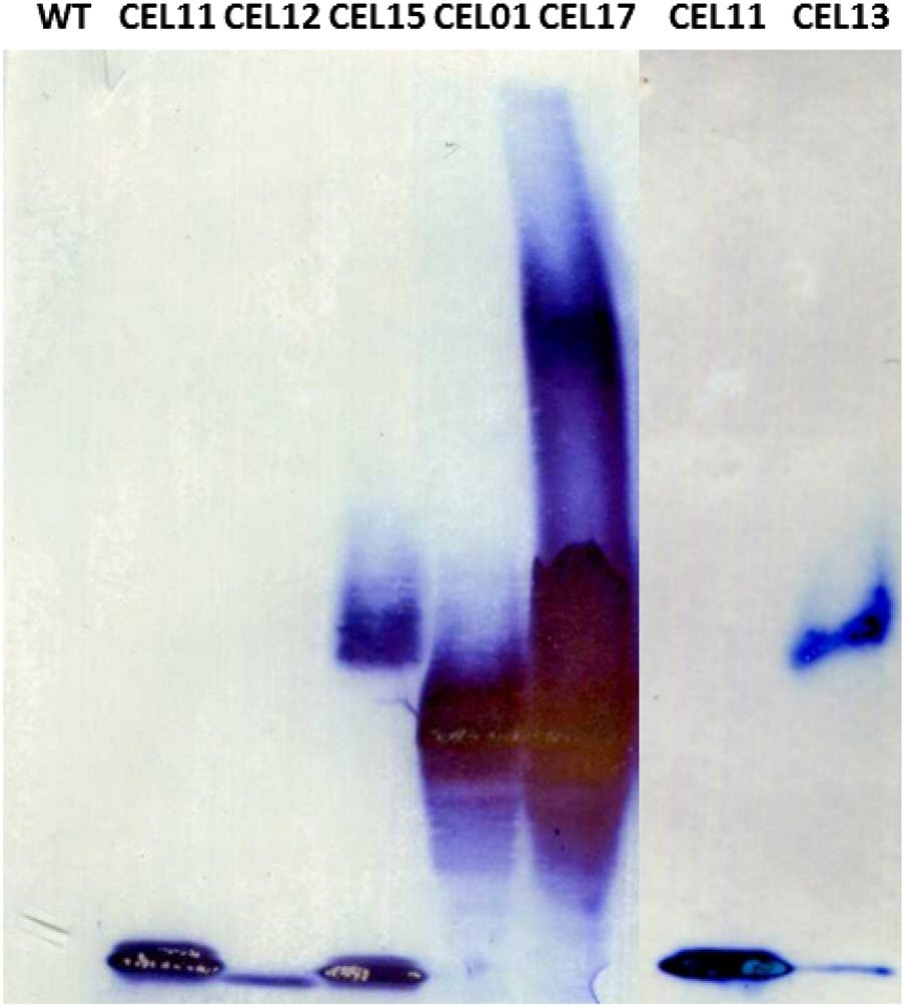
Native PAGE/western blot analysis of supernatants from recombinant *C. acetobutylicum* strains producing heterologous, FLAG-tagged cellulosome components encoded by chromosomally integrated gene constructs. Supernatants were obtained at an OD_600_ of approximately 0.8 and proteins concentrated approximately 100-fold by ultrafiltration with a 10,000 MWCO filter before being separated on 3–8 % Tris–Glycine gradient gels; after blotting, cellulosomal components were detected using ANTI-FLAG M2 monoclonal antibody-horseradish peroxidise conjugate. Supernatants were obtained from strains CEL11, expressing a gene encoding Cel9G-FLAG, CEL12, expressing a gene encoding Cel48F-FLAG, CEL13, expressing a gene encoding Xyn10A-FLAG, CEL15, expressing genes encoding all three enzymes (Xyn10A-FLAG, Cel9G-FLAG, and Cel48F-FLAG), CEL01, expressing a gene encoding the BBCipC3-FLAG miniscaffoldin, and CEL17, expressing genes encoding all three enzymes and the BBCipC3-FLAG miniscaffoldin

Native PAGE analysis (Fig. 2) revealed that the secreted components were capable of forming a cellulosome in vivo, visible as a large ‘smear’ pattern in the XGF:C sample. As no band corresponding to free Cel9G or Cel48F is visible, it is possible to infer that all the secreted enzymes are incorporated into cellulosomal complexes, and that the scaffoldin is in excess relative to the secreted enzymes. Interestingly, two ‘smears’ can be observed: one slowly migrating and indistinct, and one darker and more well defined. In preliminary work where only Cel9G and CipC3 were coexpressed, only the latter was observed (data not shown), suggesting that the indistinct ‘smear’ consists of complexes incorporating Xyn10A. This would be in agreement with the slower migration speed and less distinct band of uncomplexed Xyn10A. Interestingly, the ‘smear’ pattern observed for *C. cellulolyticum*-derived minicellulosomes does not resemble the defined banding patterns observed for *C. thermocellum*-derived complexes [29].

### Anchoring of scaffoldin to the cell wall

In the natural environment, cellulosomes are typically anchored to the host cell. This allows the direct adherence of the cell to the substrate. As a result, the cell is able to remain in close proximity to the released sugars. Thus, when grown on cellulose in fast-flow culture, *C. thermocellum* has been observed to lose only 13.7–29.1 % of the released sugars into the medium [54]. Furthermore, by removing sugars from the immediate surroundings, the effect of product inhibition on the activity of the cellulases [55] is reduced. In nature, anchoring is accomplished through a variety of methods. ‘Complex’ cellulosomes, such as those of *C. thermocellum*, are able to anchor to the cell through a cohesin–dockerin interaction with a cell wall-anchored secondary scaffoldin [21]. For ‘simple’ cellulosomes, attachment has been observed through interaction with cell wall-anchored cellulase enzymes, as seen in *C. cellulovorans* [56], or through non-specific cell wall interactions, such as in *C. cellulolyticum* [57].

One interesting mechanism of cellulosome anchoring is found in certain *Ruminococcus* species, where the secondary scaffoldin is covalently attached to the cell wall via a sortase [58, 59]. Sortases are a group of cysteine transpeptidases that are used by Gram-positive bacteria to covalently anchor proteins to the cell wall. The substrates are recognisable by the presence of a sortase signal sequence, consisting of a canonical LPXTG motif, hydrophobic region, and positively charged C-terminus [60]. The hydrophobic region and charged C-terminus lead to retention of the protein in the cell membrane, where it is cleaved by the sortase upon recognition of the LPXTG motif, forming an thioester intermediate; a nucleophilic attack from lipid II results in the formation of a lipid II-protein complex, which is integrated into the peptidoglycan [60].

The genome of *C. acetobutylicum* encodes a predicted sortase (CA_C0204) with four potential substrates [61]. As the use of the native sortase system would be a useful method for the anchoring of our own synthetic cellulosomes to the cell wall, we decided to examine the functionality of this system by appending the native sortase signals to our mini-CipC3 scaffoldin. Of the four potential substrates, we focused on an Icc-family phosphohydrolase (CA_C0205) and a cyclic AMP phosphorylase (CA_C0353). The other two proteins, an unclassified membrane protein and a SpoIID-like protein, were discounted. The membrane protein lacks an N-terminal secretion signal and true LPXTG motif, having instead an LPKSG sequence, whereas the SpoIID-like protein has multiple LPXTG motifs throughout the protein, and a much shorter hydrophobic region [61].

As sortase signal sequences are located at the C-terminus of the target proteins, we wished to replicate the same arrangement within our mini-CipC constructs. Thus, it was necessary to generate a new mini-CipC3 variant without a C-terminal FLAG tag. In initial experiments, we designed a mini-CipC variant with an N-terminal FLAG tag, located immediately after the cleavage site of the CipC secretion signal peptide; however, this was found to affect the secretion of the protein (data not shown). As a result, we chose to locate the FLAG tag internally, in the linker region between the second and third cohesins, generating the CipC2F3 miniscaffoldin. Two variants, CipC2F3-CA_C0205ss and CipC2F3-CA_C0353ss, carried the sortase signal sequences of the *C. acetobutylicum* Icc-family phosphohydrolase and cyclic AMP phosphorylase, respectively. All CipC-encoding variant genes were cloned into the pMTL-JH16 vector and integrated into the *C. acetobutylicum* genome at the *thl* locus, under the control of the genomic P_*thl*_ promoter, in the same manner as the CipC3-FLAG construct described previously.

**Fig. 3:**
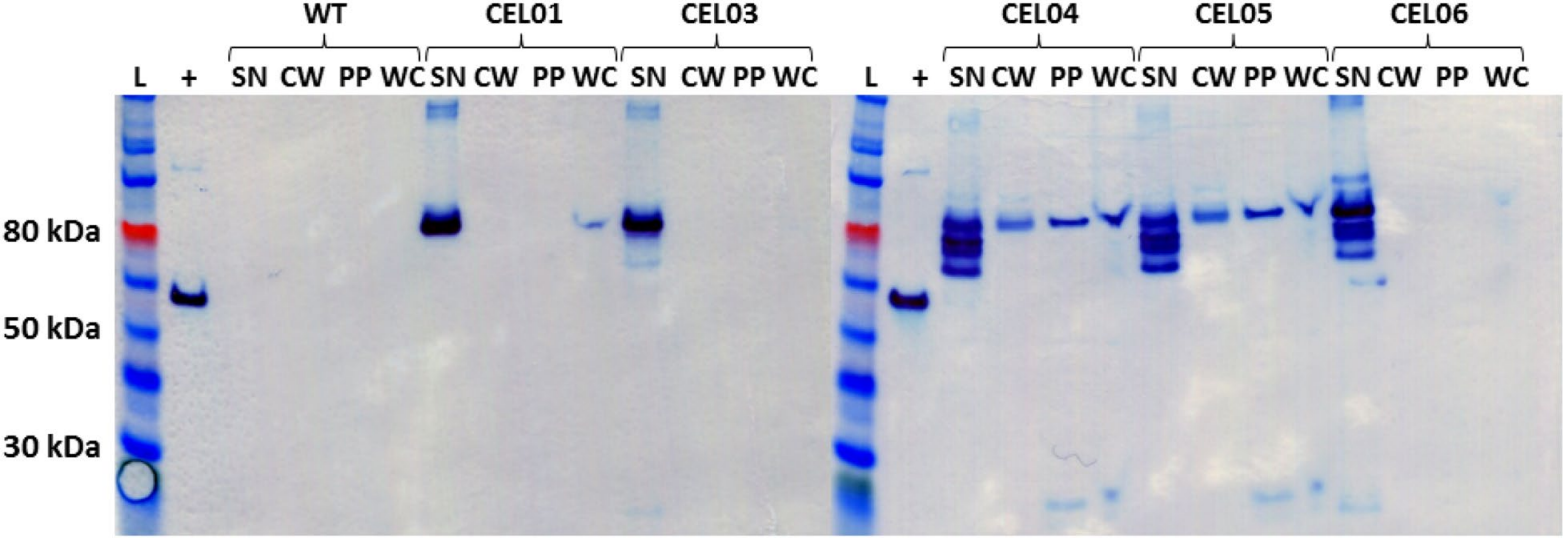
Localisation of FLAG-tagged and sortase signal-linked CipC3 miniscaffoldin variants when produced alone and when co-produced with *S. aureus* SrtA (Sa-SrtA). Cells were fractionated according to the protocol in the “Methods” section, and fractions were subjected to SDS-PAGE and western blot. Proteins were separated on 4–12 % Bis–Tris gradient gels; after blotting, cellulosomal components were detected using ANTI-FLAG M2 monoclonal antibody-horseradish peroxidise conjugate. Four fractions were examined: *SN* culture supernatant; *CW* cell wall digest; *PP* protoplast fraction; *WC* whole cell fraction. Six different strains were examined: wild-type *C. acetobutylicum* ATCC 824 (WT); CEL01, expressing a gene encoding CipC3-FLAG; CEL03, expressing a gene encoding the internally FLAG-tagged variant CipC2F3; CEL04, expressing a gene encoding CipC2F3-CA_C0353ss; CEL05, expressing a gene encoding CipC2F3-CA_C0205ss; and CEL06, expressing genes encoding CipC2F3-CA_C0205ss and Sa-SrtA. Sa-SrtA was expected to recognise the sortase signal sequence of CA_C0205, but was not expected to be able to anchor the protein to the cell wall, and correspondingly, cell wall anchoring of CipC2F3-CA_0205ss is absent in CEL06. L, ColorPlus Prestained Protein Ladder, broad range (10–230 kDa); +, carboxy terminal FLAG-BAP control protein (50 kDa)

Strains expressing the different mini-CipC gene variants were subjected to cell fractionation (Fig. 3). The introduction of the FLAG tag between the second and third cohesin did not appear to have any effect on the distribution of CipC as compared to the original C-terminally FLAG-tagged mini-CipC3. However, the introduction of the CA_C0353 and CA_C0205 sortase signal sequences resulted in small but detectable levels of CipC in the cell wall digest and cell fractions, suggesting that a small amount of CipC was being successfully anchored to the cell by the native sortase. To further confirm that this attachment was due to the presence of the sortase signal, we chose to co-express the *srtA* gene from *Staphylococcus aureus*, encoding a class A sortase (Sa-SrtA); *srtA* was introduced downstream of the CipC2F3-CA_C0205ss scaffoldin gene, forming an operon under the control of the genomic P_*thl*_ promoter. This sortase was chosen as it would be able to recognise the LPXTG motif found in the *C. acetobutylicum* sortase signals, but would be unable to subsequently attach the protein to the cell wall. Cell wall peptides are cross linked, and cell walls have been classified into different categories based on the structure of the cross link; *S. aureus* has a type A3a cell wall, with an interpeptide bridge consisting of five glycine residues, whereas *C. acetobutylicum* has been predicted to have a type A1ɣ cell wall, where there is direct cross linking between a D-Ala residue of one peptide to an m-Dpm residue of another [62]. The *S. aureus* sortase system anchors proteins to the pentaglycine bridge [63], which does not exist in *C. acetobutylicum*. As a result, while Sa-SrtA would be able to recognise and cleave the LPXTG motif, the protein would not be anchored to the cell wall, and would instead be released into the supernatant after nucleophilic attack from a water molecule [64]. Correspondingly, when Sa-SrtA and CipC2F3-CA_C0205ss are co-produced, no signal can be observed in the cell wall and cell fractions, providing further evidence that the signal seen in the cell wall and cell fractions is a result of anchoring through the native sortase system.

### Effect of overexpressing the native sortase

Although we were able to demonstrate some cell wall attachment, the amount of anchored CipC remained low compared to the amount in the supernatant. We hypothesised that the *C. acetobutylicum* sortase may either be only weakly active or poorly expressed, and that the high level of CipC3 expression was overwhelming the native system. Therefore, we decided to overproduce the native sortase (Ca-SrtA) by introducing a plasmid vector. To provide a comparison, we chose to express two other sortase genes, encoding the sortases from *Bacillus cereus* (Bc-SrtA) and *Listeria monocytogenes* (Lm-SrtA), respectively. Both organisms are predicted to share the same cell wall type as *C. acetobutylicum* [62] and both sortases recognise the same LPXTG motif [65, 66]. The sortase of *L. monocytogenes* was chosen as it has been experimentally demonstrated to anchor proteins to the cross linking m-Dpm residue [67], whereas the sortase of *B. cereus* was chosen due to its extremely high identity with the sortase of *Bacillus anthracis*, which had been recently used to anchor a synthetic miniscaffoldin to the cell wall of *B. subtilis* [14]. All three sortase genes were amplified from genomic DNA and cloned into the pMTL82151 shuttle vector under the control of the P_*Tcpf*_ promoter, comprising the P_*thl*_ of *C. perfringens* with the RBS of the *C. acetobutylicum* P_*thl*_ promoter.

**Fig. 4:**
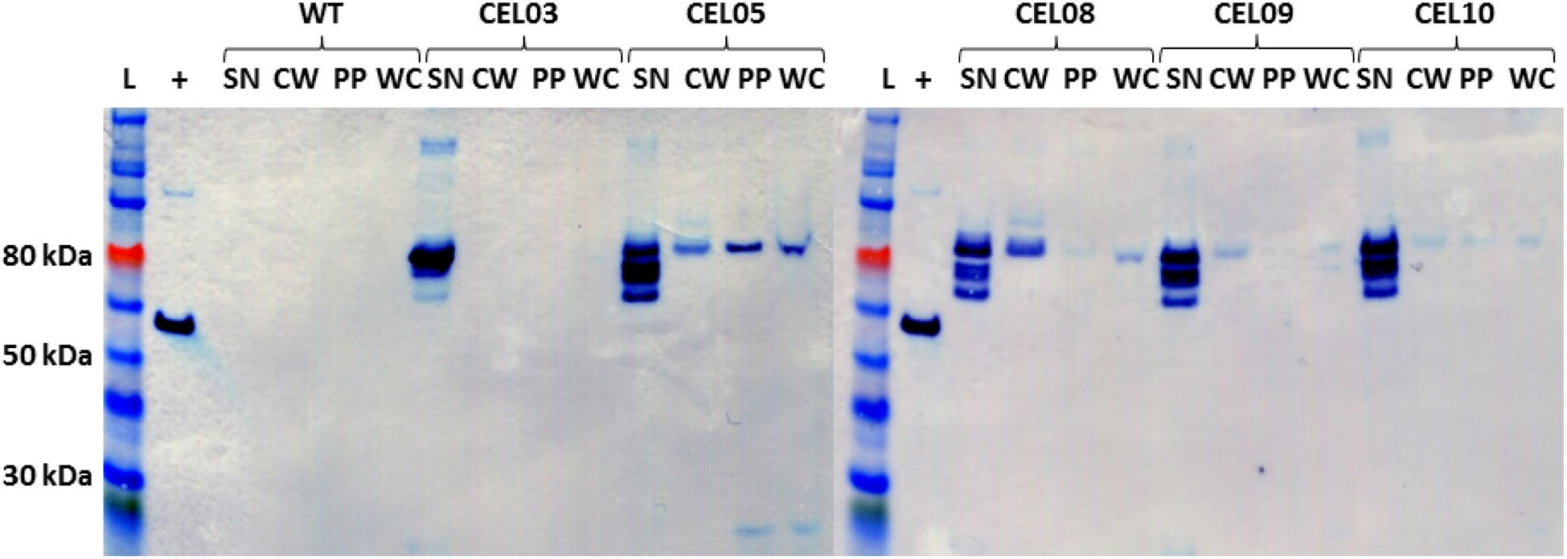
Coexpression of genes encoding sortases with a sortase signal-linked scaffoldin. Cells were fractionated according to the protocol in the “Methods” section, and fractions were subjected to SDS-PAGE and western blot. Proteins were separated on 4–12 % Bis–Tris gradient gels; after blotting, cellulosomal components were detected using ANTI-FLAG M2 monoclonal antibody-horseradish peroxidise conjugate. Four fractions were examined: S*N* culture supernatant; *CW* cell wall digest; *PP* protoplast fraction; *WC* whole cell fraction. Six different strains were examined: *C. acetobutylicum* ATCC 824 wild-type (WT); CEL03, expressing a gene encoding CipC2F3; CEL05, expressing a gene encoding CipC2F3-CA_C0205ss, and CEL08, CEL09, and CEL10, combining production of CipC2F3-CA_C0205ss with overexpression of the *srtA* genes of *C. acetobutylicum*, *L. monocytogenes*, and *B. cereus*, respectively. L, ColorPlus Prestained Protein Ladder, broad range (10–230 kDa); +, carboxy terminal FLAG-BAP control protein (50 kDa)

Combining production of CipC2F3-CA_C0205ss with overexpression of the native sortase gene resulted in an increase of CipC signal in the cell wall fraction (Fig. 4). With this level of protein, it is possible to see more clearly an additional band above the CipC band, potentially resulting from the presence of residual peptidoglycan fragments attached to the scaffoldin as has been seen with cell wall digests of *S. aureus* [68]. However, the signal in the cell fractions was significantly reduced. A likely explanation for this is that the signal in the cell fractions represents CipC that is anchored at the membrane, rather than on the cell wall; the protocol used for cell lysis would not hydrolyse significant amounts of peptidoglycan. Schneewind et al. [69] observed that when the LPXTG motif was removed from a fusion protein bearing a sortase signal, the localisation of that protein shifted from the cell wall to the membrane and cytoplasmic fractions. Accordingly, if the level of CipC production was sufficient to overwhelm the capacity of the native sortase system, we would expect a ‘backlog’ of unprocessed substrate to accumulate in these same fractions. When a sortase gene is overexpressed, this substrate can be processed, removing it from the membrane. Nevertheless, a significant amount of protein is lost to the supernatant, despite the overproduction of the sortase. Although the level of CipC in the wall fraction increases when the native sortase gene is overexpressed, this does not seem to be matched by a corresponding decrease in the full-length CipC in the supernatant, but by a decrease of the 75 kDa degradation product. Thus, it is possible that a certain proportion of CipC is able to escape directly to the supernatant when being secreted, and that CipC is lost from the cell membrane due to the action of native proteases removing the C-terminal region before the protein can be processed by the sortase. Interestingly, while *C. acetobutylicum*, *B. cereus* and *L. monocytogenes* are predicted to have the same cell wall type, the expression of genes encoding Bc-SrtA and Lm-SrtA appears to reduce the level of cell wall anchoring, although they do not completely abolish anchoring in the same way as the *S. aureus* sortase. This may be a result of the cell wall structures of *C. acetobutylicum*, *B. cereus* and *L. monocytogenes* differing in ways other than the cross-linker residues; alternatively, the more acidic conditions of a *C. acetobutylicum* culture may be suboptimal for Bc-SrtA and Lm-SrtA activity.

While the majority of sortases have been shown to anchor proteins to the outside of the cell wall, there is a possibility that the CipC protein could be localised to another region. Class C sortases have been demonstrated to covalently join together multiple pilin subunits during pilus assembly, and in *B. anthracis*, a sortase substrate has been demonstrated to be anchored to the forespore by a class D sortase [60]. The sortase of *C. acetobutylicum* does not appear to fall into the classes described by Spirig et al. [60] and it is, therefore, difficult to predict the true role of Ca-SrtA from sequence similarity alone. Furthermore, there is a possibility of that CipC is being mis-sorted. The replacement of the sortase signal of *S. aureus* protein A with other sortase signals can lead to mis-sorting even when a signal from the same organism is used [69]. Moreover, as CipC is not a native sortase substrate, then the potential for mis-sorting is likely to be even greater.

**Fig. 5:**
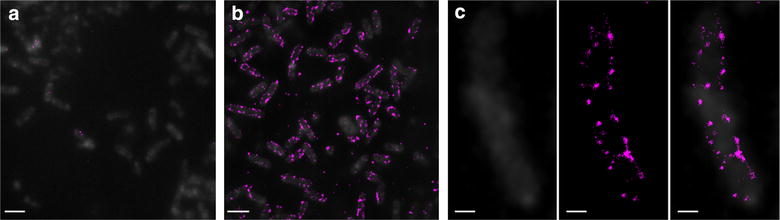
Wide-field (*grey*) and dSTORM super resolution (*magenta*) fluorescent microscopy images of CipC-expressing *C. acetobutylicum* bacteria. The bacteria were visualised by their autofluorescence, detected in the *green channel* and shown here in *grey*; CipC was labelled by addition of anti-FLAG mouse monoclonal antibody followed by anti-mouse goat antibody conjugated to Alexa Fluor 647 dye and is shown as *magenta pixels* detected as single molecules in the far *red channel*. **a** Negative control (CEL07, overexpressing the native *srtA* gene) showing few background events, see the few pixels in *magenta*. **b** *C. acetobutylicum* CEL08 bacteria (producing CipC2F3-CA_0205ss and overexpressing the native *srtA* gene) displaying high number of CipC molecules on their surface. **c** Single CEL08 bacterium; the *magenta pixels* represent single molecule localisations of the labelled CipC. *Scale bars* **a**, **b** 2 µm, **c** 0.5 µm

Although the results of the cell fractionation strongly suggest that CipC is being anchored to the cell wall, we sought further evidence through the use of fluorescence microscopy. The microscopy images obtained (Fig. 5) revealed a clear signal in cells producing the CipC2F3-CA_C0205ss miniscaffoldin and Ca-SrtA sortase (Fig. 5b), whereas those only producing the sortase (Fig. 5a) only displayed a very small amount of background signal. As the secondary antibody is not monoclonal, it is likely that this small amount of background is a consequence of the animal source of the antibody already having antibodies that recognise *C. acetobutylicum* following prior exposure to a similar *Clostridium* species. The signal can be seen to be localised at the cell wall, providing evidence for the correct localisation of the scaffoldin. This conclusion is further supported by the observation that the signal appears to form a spiral around the outside of the cell (Fig. 5c), resembling the distribution of a sortase-anchored protein observed in *B. subtilis* [70].

### Assaying the cellulolytic potential of the strain

Having demonstrated the expression of all heterologous cellulosome components, the in vivo assembly of the components into a complex, and the successful anchoring of a modified scaffoldin protein to the cell wall, we aimed to develop a strain capable of producing a functional, cell wall-anchored minicellulosome. Due to the use of both the *thl* and *pyrE* loci for strain construction, this was easily accomplished by the introduction of the CipC2F3-CA_C0205ss gene at the *thl* locus of *C. acetobutylicumpyrE*:XGF, followed by transformation with the pMTL82151_Tcpf_CaSrtA plasmid.

**Fig. 6:**
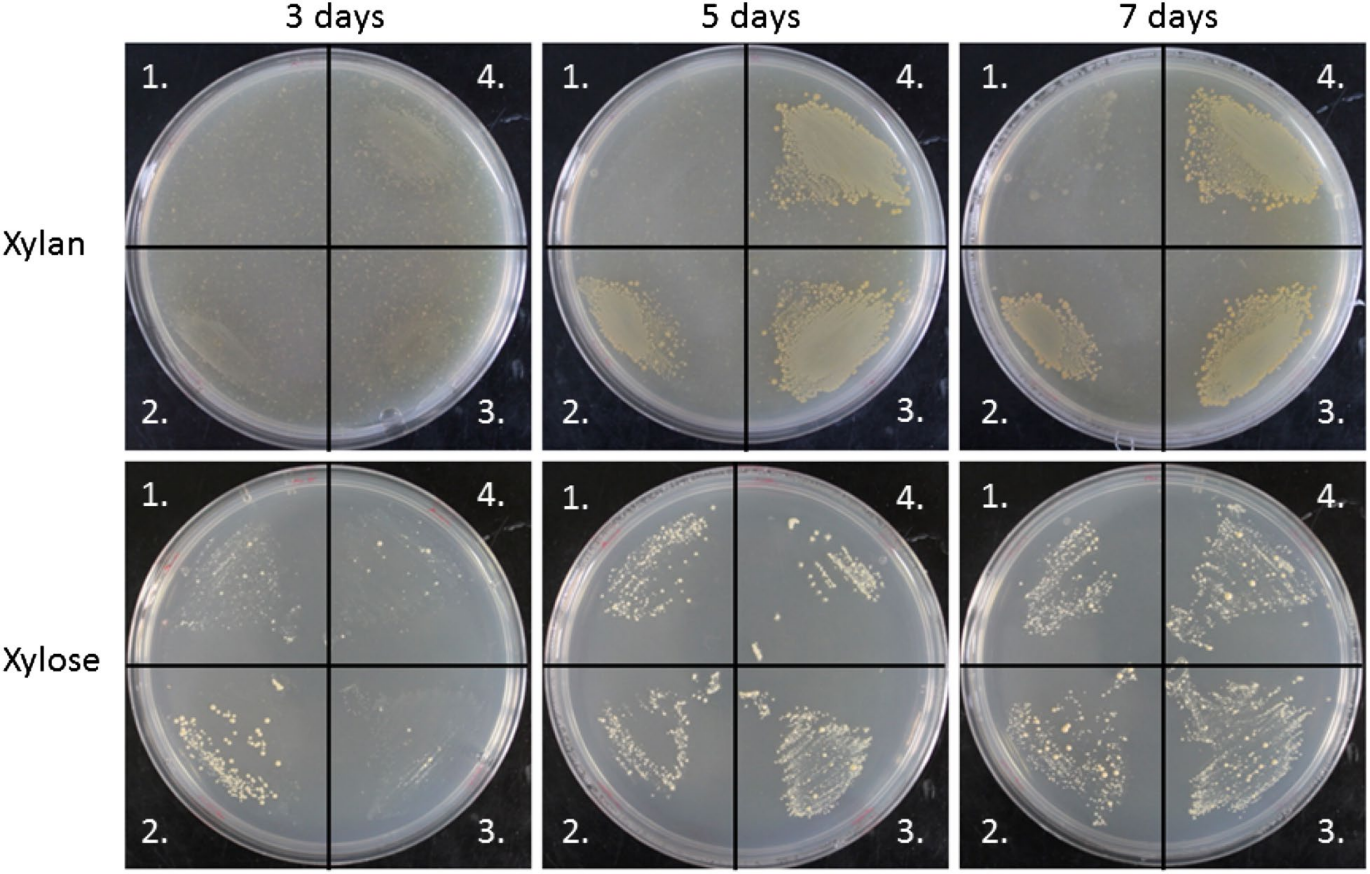
Analysis of xylan utilisation by recombinant *C. acetobutylicum* strains producing anchored and unanchored minicellulosome constructs. Strains were streaked onto plates containing 30 ml CBM-agar with 1 % of either xylose or xylan as the sole carbon source. Plates were incubated in an anaerobic cabinet at 37 °C for the indicated time and were subsequently removed for imaging. *1* CEL07, overexpressing the native *srtA* gene; *2* CEL16, overexpressing the native *srtA* gene and producing the enzymes Xyn10A-FLAG, Cel9G-FLAG, and Cel48F-FLAG, without scaffoldin; *3* CEL19, overexpressing the native sortase gene, producing all three enzymes, and producing the unanchored scaffoldin CipC2F3; *4* CEL21, overexpressing the native sortase gene, producing all three enzymes, and producing the sortase signal-bearing scaffoldin CipC2F3-CA_C0205ss

We then assayed the ability of the strains to grow on solid medium with only cellulosic substrates as a carbon source; the strains were streaked onto CBM-agar containing beechwood xylan, phosphoric acid-swollen cellulose (PASC), powdered wheat straw, or dilute acid pretreated willow bark. While no growth was observed on the cellulosic substrates (data not shown), a clear difference was visible when the recombinant strains were plated on xylan (Fig. 6). For the negative control strain, growth on xylan was only faintly visible after 7 days. However, for the strains expressing the hydrolase gene cassette, the rate of growth on xylan was largely similar to that on xylose, with growth appearing after 3 days and becoming well established at 5 days. This growth was accompanied by a visible zone of clearance, which is not apparent for the negative control even after 7 days of incubation. Interestingly, the colony phenotype of xylan-grown *C. acetobutylicum* cultures was very different from that of those grown on xylose; colonies grown on xylose were pale, with little growth into the agar, and with single colonies emerging within the streak, whereas colonies grown on xylan were golden brown in colour and were firmly embedded into the agar. The strain CEL13, expressing Xyn10A only, was also discovered to grow on xylan plates, demonstrating that expression of Xyn10A was sufficient for growth (data not shown).

During the course of this work, we discovered that the laboratory strain of *C. acetobutylicum* ATCC 824 used (ATCC 824 COSMIC) contained a deletion of several genes located on the megaplasmid, including *ptna*, *manY*/*levF*, *ptnd*, CA_P0069, CA_P0070, and CA_P0071 [71]. As CA_P0071 is predicted to encode a xylanase, it was necessary to repeat the above experiment to compare the growth of the older strain with a newer strain re-acquired from the ATCC (November 2011). However, neither strain of *C. acetobutylicum* displayed growth on xylan plates (data not shown). This result is in concordance with the literature [39] and demonstrated that the inability of our wild-type strain to grow on xylan as a sole carbon source is not solely a result of the loss of one of the native xylanases.

**Fig. 7:**
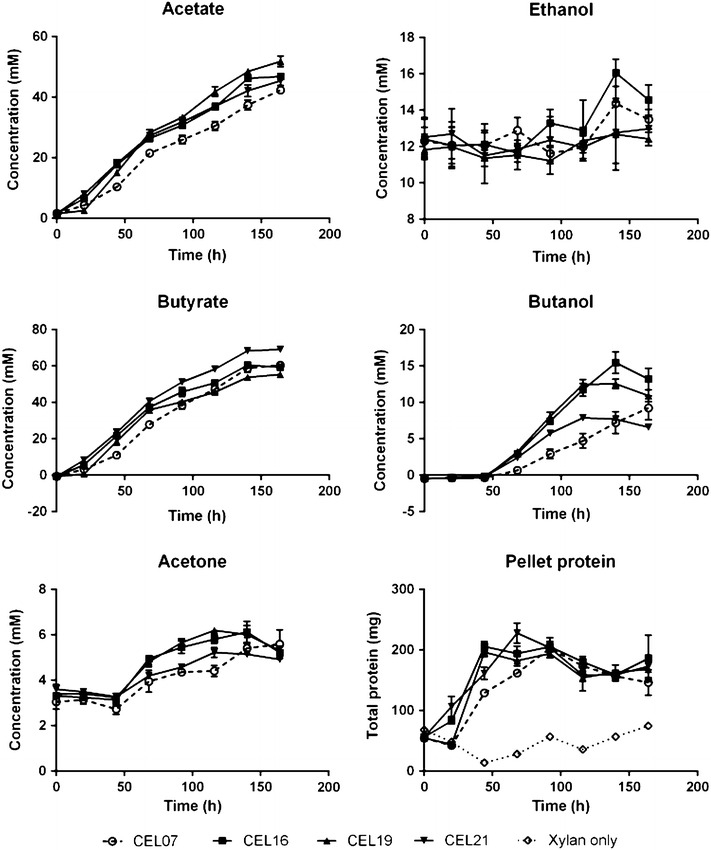
Product profiles of batch fermentations of 5 % beechwood xylan by recombinant *C. acetobutylicum* strains. Strains analysed include: CEL07, overexpressing the native *srtA* gene; CEL16, producing Xyn10A, Cel9G and Cel48F and overexpressing the native *srtA* gene; CEL19, producing the three hydrolases and the unanchored CipC2F3 scaffoldin and overexpressing the native *srtA* gene; and CEL21, producing the three hydrolases and the wall-anchored scaffoldin CipC2F3-CA_C0205ss and overexpressing the native *srtA* gene. Results represent the average of three batch cultures; *error bars* represent standard deviation. The data obtained from the fermentation have been provided in Additional file 3

To further investigate the ability of our strains to grow on xylan, we carried out open batch cultures of 5 % beechwood xylan in liquid medium. The strains were precultured in xylose, as this has been demonstrated to enable wild-type *C. acetobutylicum* ATCC 824 to grow on xylan [39], thus allowing a fairer comparison of the solvent profiles of the control and enzyme-producing strains. Under these conditions, all the strains were able to grow using the xylan as a sole carbon source (Fig. 7). Supernatant samples were then analysed via gas chromatography (GC). When grown on xylan, all the analysed strains produced organic acids as the major products, with roughly 60 mM butyric acid and 50 mM acetic acid present at the end of the fermentations. No significant re-assimilation of the acids could be observed and, accordingly, acetone production was very low. Butanol production in the negative control strain CEL07 was also low, with an average production of 9.2 mM (0.82 g/L) after 164 h. This result could be expected based on the literature; similar results have been observed for other solventogenic strains when grown on xylan as a sole carbon source, including *C. acetobutylicum* ATCC 39236 grown on larchwood or oat spelt xylan [72], *Clostridium beijerinckii* NCP 260 grown on oat spelt xylan [73], and *Clostridium* sp. G117 grown on beechwood xylan [74]. Nevertheless, in concordance with the plate growth analysis, the xylanase-expressing strains were able to grow more rapidly, with a faster accumulation of biomass (as estimated by cell pellet protein) and organic acids. Interestingly, the xylanase-expressing strains were also able to produce significantly more butanol, with the best performing strain (CEL16) producing a maximum of 15.4 mM (1.36 g/L) after 140 h. Strains CEL16 and CEL19, which produced more butanol, also showed a corresponding decline in the rate of butyrate production, relative to the control, after the onset of butanol production; this is likely to be a result of butyrate re-assimilation. Acetone production in these strains was also slightly higher, correlating with butanol production. However, ethanol could not be accurately assayed, due to the addition of antibiotics dissolved in ethanol to the culture, low detection ability through our GC method, and (presumably) a low level of production. Solvent production in these strains appeared to cease after 116–140 h, corresponding with a decrease in overall pellet protein, possibly as a result of organic acid accumulation or exhaustion of readily hydrolysable substrate. One possible explanation for the increased production of butanol in the xylanase-expressing strains could be a greater proportion of released xylose monomers or shorter xylo-oligosaccharides; when *Clostridium* sp. G117 was grown on xylose, greater amounts of solvents were produced than when xylo-oligosaccharides were fermented, with xylan giving the lowest levels of solvents and the highest level of butyrate [74]. This may also explain the lower production of butanol by strain CEL21, expressing a cell wall-anchored cellulosome; xylo-oligosaccharides released by wall-anchored Xyn10A would be more likely to be taken up by the cell, whereas in other strains, they may remain in the supernatant and undergo further digestion.

**Fig. 8:**
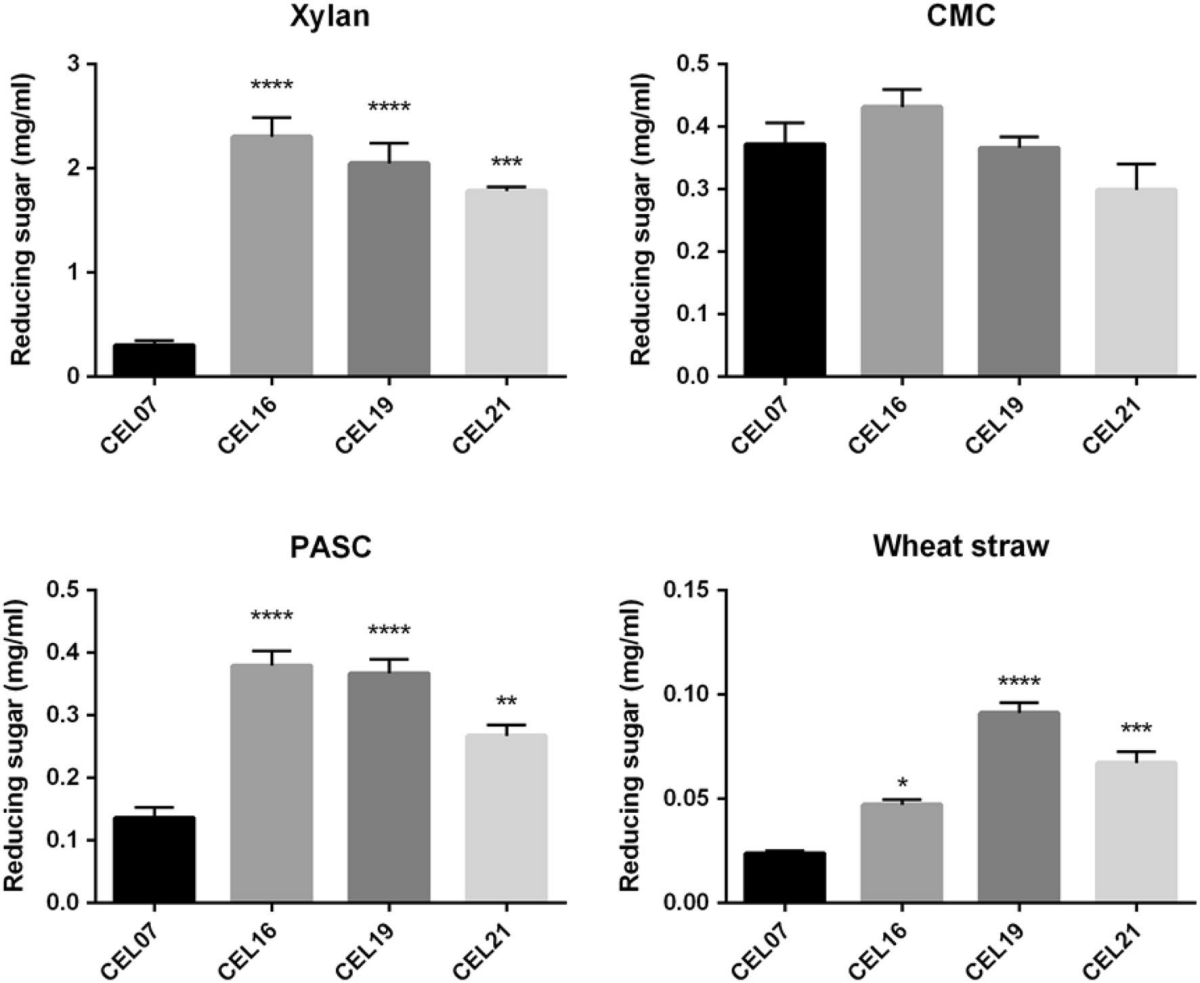
Release of reducing sugar from beechwood xylan, CMC, PASC and wheat straw by 10-fold concentrated supernatants from recombinant strains of *C. acetobutylicum*. Supernatants were collected from recombinant strains of *C. acetobutylicum* and concentrated 10-fold; concentrated supernatants were incubated with 1 % substrate and the released sugars analysed by DNS assay. The following strains were analysed: CEL07, overexpressing the native *srtA* gene; CEL16, producing Xyn10A, Cel9G and Cel48F and overexpressing the native *srtA* gene; CEL19, producing the three hydrolases and the unanchored CipC2F3 scaffoldin and overexpressing the native *srtA* gene; and CEL21, producing the three hydrolases and the wall-anchored scaffoldin CipC2F3-CA_C0205ss and overexpressing the native *srtA* gene. Differences between levels of released sugars were measured by 1-way ANOVA. Results represent the average of three technical replicates, with the DNS assay also carried out in triplicate; *error bars* represent standard error of the mean. Hydrolysis reactions were carried out for 2 h for xylan and CMC and 72 h for PASC and wheat straw. Significantly more reducing sugar was released from xylan by strains CEL16 and CEL19 (*P* ≤ 0.0001, ****) and by CEL21 (*P* ≤ 0.001, ***) when compared to CEL07. For CMC, there were no statistical differences between any of the strains. For PASC, there was a significant difference in released reducing sugar between CEL07 and the strains CEL15 and CEL19 (*P* ≤ 0.001, ***) and between CEL07 and CEL21 (*P* ≤ 0.01, **); there was also a significant difference (*P* ≤ 0.05) between CEL21 and strains CEL16 and CEL19. For wheat straw, there was a significant difference (*P* ≤ 0.05, *) between CEL07 and CEL16, between CEL07 and CEL19 (*P* ≤ 0.0001, ****) and between CEL07 and CEL21 (*P* ≤ 0.001, ***); there were also significant differences between CEL16 and CEL19 (*P* ≤ 0.001), CEL16 and CEL21 (*P* ≤ 0.05), and CEL19 and CEL21 (*P* ≤ 0.05). The data obtained from the sugar release assay have been provided in Additional file 3

Although we were unable to observe growth on lignocellulosic substrates, we decided to carry out sugar release assays to examine the potential of the strains to hydrolyse different substrates. Supernatants were obtained from the strains, concentrated 10-fold, and incubated in the presence of xylan, CMC, PASC, ball-milled wheat straw, and Avicel. After 2 h (xylan, CMC), or 72 h (PASC, wheat straw, Avicel), the concentrations of released reducing sugars were analysed by DNS (3,5-dinitrosalicylic acid) assay (Fig. 8). While no difference was observed on CMC, significant increases in reducing sugars were observed when xylan, PASC, or wheat straw was used as the substrate. The greatest difference was observed when xylan was used as the substrate, although it is important to note that glucose was used as the carbon source for growth, which has been demonstrated to reduce native xylanase expression [39]. However, this allowed us to assess more easily the contribution of Xyn10A to xylan degradation. The most effective strain for xylan degradation was CEL16. This could be expected as complex formation should not assist with xylan degradation; the CBM of CipC would not be able to recognise this substrate. Conversely, supernatants from strain CEL19 had a much greater effect on wheat straw, where CBM binding would be expected. Due to the high expression of xylanase, it may be possible that the sugar released from wheat straw is mostly xylose; however, this cannot explain the significant increases seen for PASC when the hydrolase-expressing strains were used, which would be the result of either Cel9G or Cel48F activity [30, 32]. While the formation of a cellulosome did not increase the degradation of PASC, this has been observed previously with chimeric cellulosomes based on Cel9G and Cel48F [34]. CEL21 supernatants appear to have a slightly lower activity against all substrates, which is likely to be a result of the anchoring of a portion of the cellulosomes to the cell. The lack of improvement of activity on CMC is more difficult to explain, as Cel9G should be expected to hydrolyse this substrate efficiently [32]. However, *C. acetobutylicum* has a significant native activity against CMC [75], which may be obscuring any activity from Cel9G under these conditions. When Avicel was used as the substrate, no sugar release was detectable for any of the supernatants (data not shown), reflecting the extreme recalcitrance of this highly crystalline substrate.

**Fig. 9:**
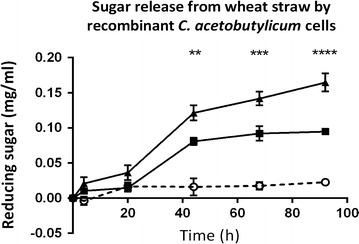
Sugar release from ball-milled wheat straw by killed cells. Washed, azide-treated cells were incubated in a suspension with 1 % (w/v) ball-milled wheat straw. Samples were taken at 4, 20, 44, 68, and 92 h and the concentrations of reducing sugars were analysed by DNS assay. Black triangles, CEL21, overexpressing the native *srtA* gene and producing a cellulosome consisting of Xyn10A-FLAG, Cel9G-FLAG, Cel48F-FLAG and the anchored scaffoldin CipC2F3-CA_C0205ss; black squares, CEL19, overexpressing the native *srtA* gene and producing a cellulosome with all three enzymes and the unanchored scaffoldin CipC2F3; open circles, wheat straw only. Results represent the average of three technical replicates with *error bars* representing standard error of the mean. Positive control was carried out using 100 μl commercial cellulase from *T. reesei* (Sigma-Aldrich) and gave a final reducing sugar concentration of 1.71 mg/ml reducing sugar at 92 h (omitted for clarity). Difference between the two *C. acetobutylicum* strains is significant to *P* ≤ 0.01 at 44 h (**), *P* ≤ 0.001 at 68 h (***), and *P* ≤ 0.0001 at 92 h (***). Results were analysed using GraphPad Prism software. The data obtained from the sugar release assay have been provided in Additional file 3

To determine whether the co-production of a cell wall-anchored scaffoldin with Xyn10A, Cel9G and Cel48F would allow a significant amount of hydrolase activity to be anchored to the cell, we carried out an additional sugar release assay on ball-milled wheat straw using azide-treated cells from strains CEL21 and CEL19, expressing cell wall-anchored and unanchored minicellulosomes, respectively (Fig. 9). Azide-killed cells were washed to remove unanchored cellulosomes, incubated in a suspension with ball-milled wheat straw over the course of 96 h, and the concentration of reducing sugars in the supernatants was assayed. We were able to detect a significant increase in released reducing sugars when the cell wall-anchored cellulosomes were expressed, as opposed to the unanchored equivalents, demonstrating the presence of functional cellulosomes on the cell surface.

This successful production of a cell wall-anchored cellulosome opens several potential avenues by which activity could be increased. Firstly, the composition of the enzymes could be further optimised; it could be possible to introduce a fourth enzyme, such as Cel9E, which is a major component of *C. cellulolyticum* cellulosomes [31] and has a strong synergy with Cel9G [34], or Cel9U, which is the most active of the *C. cellulolyticum* GH9 enzymes [33]. The use of chimeric scaffoldins and enzymes [34] could allow the generation of cellulosomes with specific compositions. Alternatively, discovery and characterisation of a greater range of promoters for use in *C. acetobutylicum* may allow an increase in the production of the various cellulosome components. Secondly, the level of cell wall attachment could be further increased. Western analysis of supernatant from strain CEL08, producing CipC2F3-CA_C0205ss and overexpressing the native sortase, demonstrates that a significant amount of scaffoldin is lost to the supernatant. Correspondingly, a significant amount of cellulase activity is lost as well, as observed from the reducing sugar and western analyses (Additional file 2: Figures S2, S3) of supernatants from strain CEL21. At present, the modified scaffoldins simply contain the sortase signal grafted to the C-terminus of the protein; the sortase signal may be more efficient in its original context, and the attachment of the C-terminal region of the original substrate may allow greater cell wall anchoring. Another possibility may be the introduction of a secondary cell wall-anchored scaffoldin at a lower level of expression. If the current high level of expression of CipC is still overwhelming the sortase system, then a secondary scaffoldin could be expressed at a lower level; a greater proportion of this scaffoldin would be bound to the cell, which would improve the ratio of anchored to unanchored minicellulosomes.

The low level of production of Cel48F is likely to be one of the main limitations in our strategy. GH48 enzymes play a crucial role in cellulolysis [76], and are generally amongst the most prevalent GH enzymes found in cellulosomes [24, 77–80]. However, *C. acetobutylicum* appears to be unable to produce large quantities of this class of enzyme; attempts at overexpressing the *C. cellulolyticum* GH48, and even the native enzyme, from plasmids have had limited success [27]. Correspondingly, in our recombinant strains, Cel48F is produced at a lower level than any of the other components despite the use of the strongest available promoter. To determine whether Cel48F was forming inclusion bodies or becoming trapped in the cell membrane, we carried out a cell fractionation on strain CEL12, expressing only Cel48F-Flag from the P_*facOid*_ promoter (Additional file 3: Figure S4). However, we were unable to detect the presence of Cel48F-Flag in any of the cellular fractions analysed. This result is in concordance with the work of Mingardon et al. [27], where Cel48F produced by recombinant *C. acetobutylicum* was detectable in the supernatant, but not in cell lysates. Alternative explanations for the poor production of Cel48F could include instability of the mRNA or degradation of the protein. Purified Cel48F has been observed to degrade, losing the dockerin domain, even when stored at 4 °C [30]; as our Cel48F protein bears a FLAG tag at the C-terminus, the loss of the dockerin would result in a protein undetectable by our anti-FLAG antibody. Addressing the level of GH48 production is likely to be the greatest obstacle towards generating a CBP strain of *C. acetobutylicum*.

The improved ability of the Xyn10A-expressing strains to grow on xylan as a sole carbon source is a particularly interesting outcome. Hemicelluloses are a major component of cellulosic biomass and are a side-product of certain lignocellulose pretreatments [81, 82]. As such, they are a potential feedstock for biofuel production. A recent study has considered the possibility of using *C. acetobutylicum* to ferment the hemicellulose fraction of pretreated lignocellulose into acetone-butanol [81]. However, this required the dilute acid hydrolysis of the xylan, an additional pretreatment step which was observed to produce inhibitory compounds which negatively impacted the fermentation profile. Although *C. acetobutylicum* ATCC 824 is able to grow on xylan in liquid culture when precultured with xylose, it is not able to grow after serial cultures with xylan as a sole carbon source [39]; this may be a regulatory issue, as a mutant that was able to continually grow on xylan could be isolated. On solid medium, it is possible that Xyn10A is able to release enough xylose to induce the production of the native xylanases, or that Xyn10A is able to complement the native xylanases and thus provide increased xylan degradation. It should be considered that while many solventogenic clostridia are able to grow on xylan as a sole carbon source, the products of fermentation are typically organic acids, and there is little or no solvent production [72–74]. However, in liquid culture, our Xyn10A-expressing strains were able to grow faster and produce butanol at up to twice the rate of the control strain. The idea that this significant improvement could be provided by the activity of a single enzyme hints at the possibility of engineering *C. acetobutylicum* for CBP of hemicellulose, an interesting avenue for further study.

## Conclusions

The generation of an organism suitable for CBP of lignocellulose is an important goal for synthetic biology, promising a more sustainable and cost-effective pathway for production of biofuels and other chemicals. In this work, we have been able to accomplish several key milestones for the development of *C. acetobutylicum* as a potential CBP chassis.

Through the use of ACE technology, we have been able to stably integrate into the genome and express genes encoding a variety of GH enzymes and a scaffoldin protein, allowing the in vivo production of recombinant minicellulosomes. This has built upon our previous work by increasing the number and range of activities of the produced hydrolases, as well as by optimising the expression levels of the genes by the use of a range of promoters. However, *C. acetobutylicum* is only able to produce small amounts of the important cellobiohydrolase Cel48F, reflecting previously observed issues with the expression of this enzyme class [27, 29]. The difficulty of production of the GH48 enzymes is likely to be the main barrier to the use of *C. acetobutylicum* in CBP.

Anchoring of the cellulosome to the cell wall has been suggested to be important for growth on lignocellulose, and we have been able to achieve this through the use of the native *C. acetobutylicum* sortase system. To our knowledge, this is the first demonstration of the activity of *C. acetobutylicum* SrtA and the first demonstration of cell surface display of a heterologous protein in this organism. Although a significant amount of scaffoldin remains in the supernatant, there are several potential routes for increasing the efficiency of anchoring, such as by the fusion of a longer C-terminal sequence from the native sortase substrate, or by the optimisation of the ion content of the growth medium [83]. With further optimisation, this ability to display proteins on the cell surface of *C. acetobutylicum* may have a number of other potential applications.

We have demonstrated the formation of minicellulosomes in vivo by our recombinant strains. Furthermore, by combining cellulase production with expression of a sortase-anchored scaffoldin, we have been able to generate a strain capable of producing a cell wall-anchored minicellulosome. Although the strains are still unable to grow on lignocellulose, significant increases in released reducing sugar from xylan, PASC and wheat straw can be observed when incubated with supernatants from our recombinant strains, and from wheat straw when incubated with cells producing a cell wall-anchored minicellulosome. Additionally, expression of Xyn10A appears to enable *C. acetobutylicum* to grow on solid medium with xylan as a sole carbon source, and to produce increased amounts of butanol when grown on xylan in liquid culture. This is a significant improvement over the wild type and may suggest that engineering of *C. acetobutylicum* for CBP of xylan could be a valid goal for future work.

In conclusion, while we were unable to generate a strain capable of growth on lignocellulose, we feel that our work provides a valuable proof of concept for development of *C. acetobutylicum* for CBP, and demonstrates the potential for future engineering of this organism.

## Methods

For a full list of plasmids, strains and oligonucleotides used in this study, see Tables S1, S2 and S3 (Additional file 5). For the sequences of the proteins and genetic components used in this study, see Additional file 6.

### Construct assembly

Plasmid construction was carried out using Fermentas FastDigest restriction enzymes and Promega T4 DNA ligase (M1804) in accordance with the manufacturer’s instructions. PCR was carried out using Thermo-Fisher Phusion DNA polymerase (F-530S). *E. coli* Top10 was routinely used as the cloning host. In this section, bolded numbers in curved brackets refer to the relevant row in Table S1.

### Vectors

As the promoters used in this study contained the *lac* operator, it was necessary to introduce the *lacI*^*Q*^ gene to the vectors to repress the promoters. The construction of the vectors **pMTL_JH16_lacIQ (68)** and **pJ201_lacIQ (27)** has been previously described [29]. The *lacI*^*Q*^ gene was excised from pMTL-JH16_lacIQ via PmeI digest and introduced to the **pMTL-JH14(75)** and **pMTL-JH12 (82)** [42] vectors by blunt-end ligation after linearising with the same enzyme, generating the **pMTL-JH14_lacIQ(76)** and **pMTL-JH12_lacIQ (83)** vectors. The P_*fac*OID_ promoter of **pMTL82254_facOid_CatP (77)** was introduced to the pMTL-JH14_lacIQ vector by NotI/NheI cloning, generating the **pMTL-JH14_lacIQ_facOID (78)** vector.

To carry out iterative ACE assembly, the long homology arm of the pMTL-JH14lac vector was replaced with a sequence comprising the first ~1500 bp of BB2fdxOID_Cel9G, amplified from **pJ201_lacIQ_BB2fdxOID_Cel9G_FLAG (56)** using the **9G_arm_fd** and **9G_arm_rev** primers and cloned into the NheI/AscI sites of pMTL-JH14_lacIQ, creating the **pBW1 (85)** vector.

### Terminator constructs

All terminator sequences were synthesised as complementary pairs of single-stranded BB2-format oligonucleotides, were annealed to form double stranded DNA, and were cloned into the pJ201_lacIQ vector **(29–35)**. A BB2-format CipA2-FLAG construct was available from the **pJ201_CipA2_FLAG (28)** vector used in our previous work [29]. BB2 cloning was used to insert CipA2 downstream of the terminator sequences, producing terminator-CipA2 constructs **(36–42)**. These were ligated into the NotI/NheI sites of **pMTL-JH16 (60)**, generating integration vectors **(61–67)**, which were subsequently integrated into the *thl* locus of *C. acetobutylicum*.

### Enzymes

Plasmids containing *C. cellulolyticum* Cel9G, Cel48F, and Xyn10A **(43, 44 and 45)** were provided by Dr. Katrin Schwarz. A FLAG tag sequence was introduced at the C-terminus by BB2 cloning, either by adding a FLAG tag derived from annealed oligonucleotides, or by ligating into the **pJ201_FLAG_2xStop vector (12)**, generating **pJ202_Cel9G_FLAG (46)**, **pJ201_Cel48F_FLAG (47)**, and **pJ202_Xyn10A_FLAG (48)**. As these genes had been designed with an NdeI restriction site overlapping the start codon, the promoter could be changed via this restriction site. The NdeI/NheI fragments of pJ202_Cel9G_FLAG and pJ201_Cel48F_FLAG were ligated into the NdeI/NheI-digested pMTL-JH14_lacIQ_facOID vector, generating **the pMTL-JH14_lacIQ_facOID_Cel9G (79)** and **pMTL-JH14_lacIQ_facOID_Cel48F (80)** vectors, which were subsequently integrated at the *pyrE* locus of *C. acetobutylicum pyrE*^−^, generating the CEL11 and CEL12 strains. The P_BB2*thl*OID_, P_BB2*fdx*OID_, and P_BB2*fac*OID_ promoters were synthesised as BB2-format fragments (**5, 6, 7**), lacking the RBS and 18 bp upstream, and were integrated in front of a BB2-format *catP* gene [**pJ201_lacIQ_CatP (49)**] bearing an RBS and immediate 12 bp upstream region from the *C. acetobutylicumthl* gene. BB2 assembly thus restored the full length of the promoter, with the 6 bp BB2 scar replacing the missing 6 bp **(50, 51, 52)**; activity of the promoters was confirmed by resistance to chloramphenicol in *E. coli*. Xyn10A_FLAG, Cel9G_FLAG and Cel48F_FLAG were placed under the control of these promoters by integrating the NdeI/NheI-digested enzyme constructs into the pJ201_RBS_CatP vector, generating **pJ201_lacIQ_BB2facOID_Xyn10A_FLAG (53)**, **pJ201_lacIQ_BB2thlOID_Xyn10A_FLAG (54)**, **pJ201_lacIQ_BB2fdxOID_Cel9G_FLAG (56)** and **pJ201_lacIQ_BB2facOID_Cel48F_FLAG (57)**. BB2facOID_Xyn10A_FLAG was inserted into the NotI/NheI sites of pMTL-JH14_lacIQ, generating **pMTL-JH14_lacIQ_BB2facOid_Xyn10A_FLAG (81)**, which was integrated into the genome of *C. acetobutylicum*. To assemble the three-enzyme gene cassette, BB2fdxOID_Cel9G_FLAG was cloned upstream of the EcoT1 terminator via BB2 cloning, producing **pJ201_lacIQ_BB2fdxOid_Cel9G_FLAG_EcoT1 (58).** This construct was subsequently inserted upstream of pJ201_lacIQ_BB2facOid_Cel48F_FLAG via BB2 cloning, generating **pJ201_lacIQ_BB2fdxOID_Cel9G_FLAG_EcoT1_BB2FacOID_Cel48F_FLAG (59)**; this construct was cloned into pMTL_JH12_lacIQ via the *Not*I/*Nhe*I restriction sites, generating **pMTL-JH12_lacIQ_GF (84)**. This vector was used to integrate the BB2fdxOID_Cel9G and BB2facOID_Cel48F genes at the *pyrE* locus of *C. acetobutylicum*, generating CEL14. Similarly, the BB2thlOid_Xyn10A_FLAG construct was cloned upstream of the TtyrS terminator, generating **pJ201_lacIQ_BB2thlOid_Xyn10A_FLAG_TtyrS (55)**. BB2thlOID_Xyn10A_TtyrS was cloned into the pBW1 vector using the NotI/NheI sites, generating **pBW1_ BB2facOID_Xyn10A-FLAG_TtyrS (86)**, which was integrated at the *pyrE* site of CEL14, creating the CEL15 strain.

### CipC miniscaffoldin variants

CipC3 was assembled from three BB2-format fragments synthesised by Biomatik, the first encoding the native RBS, CBD, X domain, and first cohesin of *C. cellulolyticum* CipC [**pBMH_CBD_X_C1 (1)]**, the second encoding the second cohesin [**pBMH_C2 (2)]**, and the third encoding the third cohesin [**pBMH_C3 (3)]**. Iterative BB2 cloning was used to assemble the three fragments and introduce a FLAG tag at the C-terminus. This process generated the intermediate constructs **pJ201_CipC_CBD_X_C1_C2 (14)** and **pJ202_CipC_CBD_X_C1_C2_C3 (15)**. Additionally, a ‘scarless’ variant, **pBMH_ScarlessCipC (4)**, was synthesised as a single BB2 fragment. Both CipC3 variants were FLAG-tagged by BB2 cloning, generating **pJ201_BBCipC3_FLAG (16)** and **pJ202_ScarlessCipC3_FLAG (17)**.

In previous work, an N-terminally FLAG-tagged CipC3 variant (NCipC) had been assembled; sortase signals (CA_C0353ss and CA_C0205ss) were synthesised as BB2-format fragments **(8 and 9)** and integrated at the C-terminus through BB2 cloning, generating **pBMH_NCipC_CA_C0353ss (18)** and **pBMH_NCipC_CA_C0205ss (19)**. **pJ204_NCipC_stop (20)** was assembled by BB2 cloning into **pJ204_2xStop (13)**. Internally FLAG-tagged variants were subsequently assembled through PCR of these variants with the primers C2F3FD and C2F3_BMHrev (for constructs in pBMH) or C2F3_204rev (for constructs in pJ204), followed by BB2-format assembly of the PCR products and CipC_CBD_X_C1_C2. This generated the **pJ201_CipC2F3_CA_C0353ss (21)**, **pJ201_CipC2F3_CA_C0205ss (22)**, and **pJ201_CipC2F3_stop (23)** constructs.

All CipC3 variants were cloned into the pMTL-JH16 ACE integration vector (generating **69, 70, 71, 72, and 73**) and integrated into the *thl* locus of *C. acetobutylicum*.

### Sortases

*S. aureussrtA* was amplified using the primers SrtA_start_fd and SrtA_nostop_rev. This yielded a 635 bp fragment containing the *srtA* gene with no stop codon, a 5′ NdeI restriction site overlapping the start codon, and a 3′ NheI restriction site. An RBS from the *C. acetobutylicumthl* gene was added using the NdeI site, producing the **pJ202_RBS_SaSrtA_nostop (24)** vector. The EcoRI/NheI fragment of this vector was ligated into the EcoRI/SpeI-digested pJ204_2xStop vector, producing the **pJ204_RBS_SaSrtA_stop(25)** vector; the EcoRI/NheI fragment of pJ201_CipC2F3_CA_C0205ss was ligated into the EcoRI/SpeI-digested pJ204_RBS_SrtA_stop vector to generate **pJ204_CipC2F3_CA_C0205ss_SaSrtA (26)**. The NotI/NheI fragment of this vector was ligated into pMTL-JH16 to generate **pMTL-JH16_ CipC2F3_CA_C0205ss_SaSrtA (74)**, allowing integration of the CipC2F3_CA_C0205ss/Sa-SrtA operon into the *C. acetobutylicum* genome at the *thl* locus via ACE.

The promoter of the thiolase gene from *C. perfringens* was synthesised by Biomatik as a BB2 fragment with no RBS [**pBMH_Tcpf_noRBS (10)**] and placed upstream of a BB2-format CatP gene in the same manner as the P_BB2*thl*Oid_, P_BB2*fdx*Oid_ and P_BB2*fac*Oid_ promoters described previously. This produced the **pJ201_lacIQ_Tcpf_CatP** vector **(87)**, of which the NotI/NheI fragment was ligated into **pMTL82151 (88)** producing the **pMTL82151_Tcpf_CatP** expression vector **(89)**. *C. acetobutylicum* and *B. cereus* sortase genes were amplified from *C. acetobutylicum* ATCC 824 and *B. cereus* ATCC 10987 genomic DNA using the primers CaSrtA_fd/CaSrtA_rev and Bcer_fw/Bcer_rev, respectively. These fragments were treated with NdeI/NheI and ligated into the NdeI/NheI-digested pMTL82151_Tcpf_CatP vector, generating **pMTL82151_Tcpf_CaSrtA (90)** and **pMTL82151_Tcpf_BcSrtA (92)**. *L. monocytogenes srtA* was amplified from genomic DNA using the primers Gb_Lm_Fw and Gb_Lm_Rev; the pMTL82151_Tcpf_RBSthl_CatP backbone was digested with NdeI and NheI, and the **pMTL82151_Tcpf_LmSrtA** vector **(91)** was assembled though Gibson assembly using Gibson Assembly^®^ Master Mix (New England Biolabs) in accordance with the manufacturer’s protocol.

### Culture conditions

*E. coli* Top 10 was used for routine cloning and assembly of plasmid constructs. For electroporation of *C. acetobutylicum*, DNA was methylated by electroporation into and isolation from *E. coli* pAN-2 [84]. All *E. coli* strains were cultured in liquid lysogeny broth (LB) medium at 37 °C with shaking at 200 rpm, or on solid LB-agar at 37 °C. However, the cloning of promoter-enzyme and promoter-sortase constructs required the strains to be grown at 30 °C to maintain the stability of the constructs. *C. acetobutylicum* ATCC 824 and all derived strains were cultured inside a MACS-MG-1000 anaerobic workstation (Don Whitley) with an atmosphere of 80:10:10 (vol:vol:vol) N_2_:H_2_:CO_2_, 70 % humidity, and a temperature of 37 °C. Strains were routinely cultured on CGM-agar [85] and in liquid 2xYTG medium (16 g/l tryptone, 10 g/l yeast extract, 4 g/l NaCl, pH 5.2). Transformation of *C. acetobutylicum* was accomplished by electroporation as described previously [84]. Antibiotic selection was carried out using either thiamphenicol (15 µg/ml) or erythromycin (50 µg/ml) as appropriate. Strains in which the *pyrE* gene had been truncated were grown in the presence of 20 µg/ml uracil; *pyrE* mutants were selected on 400 µg/ml fluoroorotic acid and 1 µg/ml uracil.

### Expression of cellulosomes for western blot analysis

*C. acetobutylicum* strains were cultured for protein expression analysis in 2xYTG medium buffered to pH 7 with 40 mM MOPS as previously described [29]. *C. acetobutylicum* strains were plated on CGM-agar supplemented with the relevant antibiotics and incubated in the anaerobic cabinet overnight. In the morning, growth from these plates was used to inoculate 5 ml anaerobic 2xYTG pH 5.2; after 4 h, 5 ml 2xYTG + 40 mM MOPS pH 7 was added. After a further 4 h of growth, this culture was used to prepare a series of 10-fold serial dilutions and incubated in the cabinet overnight.

Overnight cultures in the exponential phase of growth were used to inoculate 30 ml 2xYTG + 40 mM MOPS pH 7 to an OD_600_ of 0.05; these cultures were incubated in the anaerobic cabinet until the OD_600_ had reached approximately 0.8. At this point, the entire culture was centrifuged at 5000*g* for 10 min at 4 °C; pellets were discarded, and the supernatants concentrated for western analysis. For SDS-PAGE, 20 ml supernatant samples were concentrated 100-fold via TCA precipitation as described by Schwarz et al. [86] and resuspended in 2 × loading dye + DTT (1:1 dilution of Thermo-Fisher NuPAGE^®^ LDS Sample Buffer (4X) with 1 M Tris pH 8, with DTT added to a concentration of 0.2 M). For native PAGE, supernatants were treated by addition of Proteinase Inhibitor Cocktail VII (Calbiochem) at a dilution of 50:1, and concentrated 100-fold using Corning^®^ Spin-X^®^ UF concentrators (6 ml, 10,000 MWCO) at 4000*g* in a swing bucket rotor.

### SDS-PAGE and western blotting

For denaturing PAGE, 20 µl TCA-precipitated sample in 2x loading dye was loaded onto a polyacrylamide gel (NuPAGE^®^ Novex^®^ 4–12 % Bis–Tris Protein Gels, 1.0 mm, Thermo-Fisher). Gel electrophoresis was carried out at 120 V for approximately 2 h using 1x MES SDS running buffer (diluted from NuPAGE^®^ MES SDS Running Buffer (20X), Thermo-Fisher).

For native PAGE, 10 µl Novex^®^ Tris–Glycine Native Sample Buffer (2X) (Thermo-Fisher) was added to 10 µl concentrated supernatant, and 20 µl was loaded onto a polyacrylamide gel (NuPAGE^®^ Novex^®^ 3–8 % Tris–Acetate Protein Gels, 1.0 mm). Gel electrophoresis was carried out at 150 V for approximately 2 h using 1x Tris–glycine native running buffer (diluted from Novex^®^ Tris–Glycine Native Running Buffer (10X), Thermo-Fisher).

SDS-PAGE gels were stained with Coomassie Blue; gels were washed with water 3 times for 5 min to remove SDS, and were stained by immersion in staining solution (0.1 % Coomassie Blue R, 40 % ethanol, 10 % acetic acid) for 1 h. Gels were then destained by immersion in destaining solution (40 % ethanol, 10 % acetic acid).

Western blotting was carried out using BioRad TransBlot^®^ Turbo™ transfer system and Trans-Blot^®^ Turbo™ Mini PVDF Transfer Packs in accordance with the manufacturer’s protocol, using the pre-programmed ‘mixed MW’ program (1.3 A, 25 V, 7 min). Membranes were blocked by addition of 30 ml blocking buffer, consisting of TBS (Tris-buffered saline; 50 mM Tris, 150 mM NaCl, pH 8) + 5 % milk powder (Marvel Original Dried Skimmed Milk Powder), and incubated at room temperature on a shaker at 60 rpm for 1 h. Blocked membranes were labelled by the addition of monoclonal ANTI-FLAG M2-peroxidase antibody (Sigma-Aldrich) at a concentration of 5 µl in 30 ml blocking buffer (approx. 0.167 mg/ml) and incubated overnight at 4 °C on a shaker at 60 rpm. Labelled membranes were washed six times with TBS + 0.1 % Tween 20 and stained by the addition of 3, 3′, 5, 5′-tetramethylbenzidine.

### Cell fractionation

Fractionation of *C. acetobutylicum* cells was carried out according to the method of Wilcox et al. [87], and modified in accordance with the *Clostridium saccharobutylicum* NCP 262 protoplasting method described by Allcock et al. [88]. As described above, strains were plated on CGM-agar supplemented with the relevant antibiotics, and incubated at 37 °C anaerobically for 24 h; a loop of agar was used to inoculate 10 ml reduced 2xYTG, and 10-fold serial dilutions were prepared. Actively growing overnight culture was used to inoculate 10 ml 2xYTG + 0.4 % glycine to an OD_600_ of 0.05. At an OD_600_ of 0.7–0.9, cells and supernatant were harvested for fractionation. A volume of culture equivalent to an OD_600_ of 1 in 1 ml was centrifuged at full speed in a benchtop centrifuge; the supernatant was discarded, and the pellet (the whole cell fraction) stored at −20 °C. Additionally, a volume of culture equivalent to an OD_600_ of 5 in 1 ml was centrifuged at 5000×*g* for 10 min. A sample of 1 ml supernatant (the supernatant fraction) was retained and concentrated via TCA precipitation to a final volume of 100 µl, whereas the cell pellet was washed once with 5 ml pre-reduced lysis buffer (TBS with 25 mM CaCl_2_, 25 mM MgCl_2_, and 0.3 M sucrose) and resuspended in 5 ml of the same buffer supplemented with 3 mg/ml lysozyme. After 3 h of incubation in the anaerobic cabinet, 1 ml of the lysozyme-digested cell suspension was removed and centrifuged for 3 min at 5000×*g* in a microcentrifuge pre-chilled to 4 °C. The supernatant was transferred to a fresh 2 ml microcentrifuge tube and centrifuged at full speed for 20 min. The supernatant from this centrifugation (the cell wall fraction) was concentrated to 100 µl via TCA precipitation, while the pellet (the protoplast fraction) was frozen at −20 °C. The protoplast and whole cell fractions were resuspended in 100 µl 2× loading dye + DTT, as this was observed to be sufficient for lysis. All fractions were then subjected to SDS-PAGE and western blotting as described above.

### Fluorescence microscopy

Overnight cultures of *C. acetobutylicum* strains were prepared in 2xYTG as previously described and used to inoculate 10 ml of 2xYTG medium to an OD_600_ of 0.05. At an OD_600_ of 0.8–1.0, a volume equivalent to 5 ml at an OD_600_ of 1 was harvested by centrifugation at 7000×*g* for 10 min. The resulting pellet was washed twice with 5 ml TBS and fixed with 5 ml 4 % paraformaldehyde in PBS (Alfa Aesar) for 30 min at 4 °C. After fixing, cells were washed three times with 5 ml TBS, and then blocked for 1 h in 5 ml TBS + 0.1 % sodium azide + 1 % BSA. Cells were stored in this buffer at 4 °C until required.

Blocked cells were centrifuged and labelled with Monoclonal Anti-FLAG M2^®^ (mouse) antibody (Sigma-Aldrich, F1804) at a 1:100 dilution in 1 ml TBS + 1 % BSA. Cells were incubated with this antibody for 1–2 h at room temperature on a rocker, and washed three times with 1 ml TBS + 0.1 % Tween-20. Cells were then labelled overnight with an anti-mouse Alexa-fluor^®^647-conjugated (goat) antibody (Cell Signalling, #4410) at a dilution of 1:1000 in 1 ml TBS + 1 % BSA. The labelled cells were subsequently washed overnight, resuspended in Vectashield hardening mounting medium (Vectorlabs H-1400) supplemented with 10 mM MEA (Cysteamine Hydrochloride, Sigma M6500-25G), and spotted onto a microscope slide for analysis. After the hardening of the mounting media (1 h), dSTORM imaging was performed as follows.

Super resolution (dSTORM) microscopy was carried out on a Zeiss Elyra PS.1 microscope equipped with Zen 2012 acquisition and processing software and fitted with an Objective alpha Plan-Apochromat 100×/1.46 Oil DIC M27 objective lens (Zeiss, 420792-9800-000). Samples were excited using the 642 and 405 nm laser lines (at 50.0 and 0.0700 % intensity, respectively) and fluorescence was detected using the LP 655 filterset. Imaging frequency was set to 50 Hz at a camera gain of 200.

Image processing was carried out in Zen 2012 Black using the PALM module. The 30,000 images were processed where single molecule events were identified with the peak intensity to noise value set to six. Drift correction was applied using model-based automatic, eight segments settings. To avoid oversampling the same molecule, Group filter was set to: max on time 5, off gap 10, capture radius 2, equiv. of 20 nm. Other filters applied: localisation precision 2–40 nm, number of photons 350–5000, PSF width 117–222 nm, background 40–5000.

### Plate growth assay

All strains used in the plate growth assay were cultured overnight on CGM-agar supplemented with the relevant antibiotics. Growth from these plates was used to inoculate CBM agar [89] plates supplemented with 1 % xylose or 1 % beechwood xylan (Sigma-Aldrich, X4252) instead of glucose. Plates were inoculated in triplicate, and single plates were removed from the cabinet for imaging at 3, 5 and 7 days.

### Fermentation of xylan

For growth on xylan, strains were precultured in 30 ml CBM medium with 0.5 % xylose instead of glucose to facilitate native xylanase production [39]. At an OD_600_ of 0.2–0.3, 5 ml preculture was used to inoculate three 250 ml flasks containing CBM with 5 % beechwood xylan (Sigma-Aldrich, X4252) instead of glucose and with 0.5 % calcium carbonate. An additional flask was prepared to provide a non-inoculated control. Cultures were incubated in an anaerobic chamber at 37 °C, and 1 ml samples were taken at 0, 20, 44, 68, 92, 116, 140, and 164 h after inoculation. Samples were centrifuged at full speed in a bench-top centrifuge for 3 min at 4 °C, and the supernatants and pellets were frozen.

### Solvent analysis via gas chromatography

A stock solution was prepared, containing 1 M acetone, 1 M ethanol, 1 M butanol, 1 M butyric acid and 1 M acetic acid. The stock solution was subsequently diluted to give a range of standards from 1 to 150 mM. ELGA water was used to provide a negative control. For each standard and supernatant sample, 500 µl was transferred to a microcentrifuge tube and acidified by addition of 5 µl of 10 M sulphuric acid. 500 µl propyl propionate with 50 mM valeric acid was added to each tube; tubes were vortexed, centrifuged in a bench-top centrifuge for 1 min, and 300 µl of the organic phase transferred to a glass sample vial. Solvent concentrations were analysed using GC (Thermo Scientific FOCUS GC) fitted with a Thermo Scientific Trace TR_FFAP column (30 m × 0.25 mm × 0.25 µm). A volume of 1 µl was injected into the column using a split/splitless injector at 240 °C, with a split ratio of 50:1 and a split flow of 50 ml/min. Initial oven temperature was 50 °C, maintained for 1 min, before increasing by 40 °C/min to 210 °C, and holding at 210 °C for 1 min; hydrogen was used as the carrier gas, with a constant column flow rate of 0.8 ml/min. Solvents were detected with a flame ionisation detector operated at 270 °C. Data were analysed using Microsoft Excel and GraphPad Prism software.

### Protein analysis of cell pellets

Frozen cell pellets were resuspended in 250 µl PBS and allowed to autolyse for 150 min at room temperature. Suspensions were centrifuged at full speed for 1 min and the supernatants transferred to fresh microcentrifuge tubes. Protein concentrations were analysed by Sigma-Aldrich Bicinchoninic Acid Protein Assay Kit (BCA1) according to the manufacturer’s instructions, using 96-well plates. Samples were measured in triplicate and concentrations determined by comparison to a BSA standard curve.

### Sugar release assay using concentrated supernatants

Relevant strains were grown in triplicate to an OD of 1.0 in CBM medium containing 40 mM MOPS pH 6.8, 0.5 % glucose, and supplemented with 15 µg/ml thiamphenicol. The supernatant was collected by centrifugation at 5000*g* for 10 min at 4 °C. The supernatant was subsequently concentrated 10 times using Corning Spin-X UF 20 ml 10 K MWCO columns (Sigma-Aldrich, 431488) using a swing bucket rotor at 4000 g at 4 °C. Residual sugars were subsequently removed using the Spin-X UF columns by three rounds of dialfiltration using 10 ml of 100 mM Tris–HCl, 1 mM CaCl_2_, pH 6.0.

The concentrated supernatants were incubated at 37 °C in a 1:1 ratio with a 2 % solution of substrate in 100 mM Tris–HCl, 1 mM CaCl_2_, pH 6.0, giving a final substrate concentration of 1 %. The following substrates were tested: sodium carboxymethyl cellulose (Sigma-Aldrich, 419303), beechwood xylan (Sigma-Aldrich, X4252), Avicel PH-101 (Fluka Biochemicals, 11366), PASC (prepared from Avicel PH-101 using the method described by Zhang et al. [90]), and ball-milled wheat straw. In accordance with the protocol described by King et al. [91], reactions with xylan and CMC were incubated for 2 h, whereas reactions with Avicel, PASC and wheat straw were incubated for 72 h. Samples were then analysed via DNS assay; Avicel, PASC and wheat straw samples were centrifuged at 10,000*g* for 1 min prior to analysis.

### Sugar release assay using killed cells

A stock suspension of 10 % ball-milled wheat straw in dH_2_O was prepared and sterilised by autoclavation. Sample tubes were prepared by the addition of 1 ml wheat straw suspension to 4 ml assay buffer (20 mM Tris–acetate, pH 6.0, 1 mM CaCl_2_, 0.1 % sodium azide).

Serially diluted overnight cultures of *C. acetobutylicum* strains were prepared in 2xYTG + 40 mM MOPS pH 7 as described in “Expression of cellulosomes for western blot analysis” section, except that the overnight cultures were grown in a volume of 30 ml. After overnight incubation, cultures with an OD_600_ of 0.8–1.0 were used to inoculate flasks containing 300 ml 2xYTG + 40 mM MOPS pH 7 to an OD_600_ of 0.05. These cultures were grown to an OD_600_ of 0.8–1.0. At this point, a volume with an OD_600_ equivalent to an OD_600_ of 1 in 40 ml was centrifuged, and the cells washed once in 40 ml assay buffer before being resuspended in 40 ml assay buffer.

Sample tubes were treated by addition of 5 ml cell suspension and incubated at 37 °C with shaking at 200 rpm. Each condition was prepared in triplicate. A positive control was carried out by the addition of 100 µl *Trichoderma reesei* cellulase preparation (Sigma-Aldrich, C2730) to tubes containing 10 ml assay buffer with 1 % wheat straw. Samples were taken at 0, 12, 20, 44, 68 and 92 h, centrifuged, and the supernatants retained for analysis.

### Analysis of reducing sugars via DNS assay

Concentrations of reducing sugars were analysed by dinitrosalicylic acid (DNS) assay as described by King et al. [91]. Supernatant samples (60 µl) were mixed with 120 µl DNS reaction mixture (1 % dinitrosalicylic acid, 0.2 % phenol, 0.05 % sodium sulphite, 1 % sodium hydroxide, 10 % sodium potassium tartrate) and heated at 95 °C for 5 min in a 96-well PCR plate. The reaction was immediately quenched by cooling to 4 °C. Of this reaction mixture, 36 µl was diluted with 160 µl dH_2_O, and absorbance at 540 nm measured using a TECAN plate reader, with the exception of powdered wheat straw, where 100 µl reaction mix was measured without dilution, and compared to an equivalent calibration curve. All samples were measured in triplicate and results were expressed as an average of three means; measurements were normalised against the relevant blank and calibrated by comparison to glucose. Statistical analyses were carried out using GraphPad Prism software to calculate standard error of the mean (SEM) and statistical significance.

## Additional files

10.1186/s13068-016-0526-x Analysis of terminator strengths in *C. acetobutylicum* ATCC 824. This file contains Figure S1, comprising the western blot analysis of efficiencies of a range of Rho-independent terminators in *C. acetobutylicum*.
10.1186/s13068-016-0526-x SDS-PAGE/western blot of supernatants for sugar release assays. This file contains Figure S2 and Figure S3, comprising the SDS-PAGE/western blot and Coomassie stain analysis of the supernatants used in the sugar release assay shown in Fig. 8.
10.1186/s13068-016-0526-x Fractionation of strains expressing Cel48F-Flag and Xyn10A-Flag. This file contains Figure S4, comprising the western blot analysis of cell fractions of strains expressing Cel48F-Flag and Xyn10A-Flag.
10.1186/s13068-016-0526-x Fermentation and sugar release assay data. This file contains the raw data from the xylan fermentation represented in Fig. 7 and the sugar release assays represented in Figs. 8 and 9.
10.1186/s13068-016-0526-x Plasmids, strains, and oligonucleotides used in the study. This file contains Table S1, Table S2 and Table S3, containing a full list of vectors used in this study, the details of the *E. coli* and *C. acetobutylicum* strains used in this study, and a full list of all oligonucleotides, respectively.
10.1186/s13068-016-0526-x Sequences of the proteins and genetic components used in the study. This file contains the amino acid sequences of the proteins used in this study, as well as the nucleotide sequences of the promoters and terminators used in the construction of the XGF gene cassette and sortase expression vectors.

## Abbreviations

ABE: acetone-butanol-ethanol
ACE: allele-coupled exchange
BB2: BioBrick 2
BCA: bicinchoninic acid
CBM: clostridial basal medium/carbohydrate binding module
CBP: consolidated bioprocessing
CGM: clostridial growth medium
DNS: 3,5-dinitrosalicylic acid
GC: gas chromatography
PASC: phosphoric acid-swollen cellulose
PBS: phosphate-buffered saline
SEM: standard error of the mean
TBS: tris-buffered saline
TCA: trichloroacetic acid
