# Supplementary material for: Production of a functional cell wall-anchored minicellulosome by recombinant Clostridium acetobutylicum ATCC 824
Source: Biotechnol Biofuels. 2016 May 23;9:109. doi: 10.1186/s13068-016-0526-x (PMC4877998; doi:10.1186/s13068-016-0526-x)
Supplement: Supplementary file 1 — 10.1186/s13068-016-0526-x Analysis of terminator strengths in C. acetobutylicum ATCC 824. This file contains Figure S1, comprising the western blot analysis of efficiencies of a range of Rho-independent terminators in C. acetobutylicum. [file 13068_2016_526_MOESM1_ESM.docx]

**Additional File 1: Analysis of terminator strengths in *C. acetobutylicum* ATCC 824**

In order to develop the hydrolase gene cassette described in this work, it was necessary to identify terminators that would be effective in *C. acetobutylicum* ATCC 824. A selection of Rho-independent terminators were cloned upstream of the *cipA2* gene, encoding a 2-cohesin miniscaffoldin from *C. thermocellum* [29]. These terminator-gene constructs were integrated at the *thl* locus of *C. acetobutylicum*, forming synthetic operons driven by the genomic *thl* promoter.


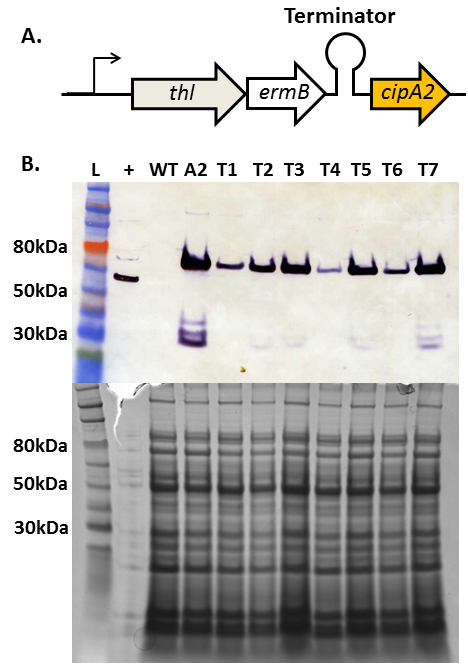


***Figure S1: Western analysis of different Rho-independent terminators in C. acetobutylicum****. Section A: Diagram showing the gene arrangement at the thiolase locus after integration. Section B: Western blot (above) and Coomassie-stained gel (below) of TCA-precipitated supernatants from wild-type C. acetobutylicum ATCC 824 (WT) and from strains integrating CipA2 with no terminator (A2) or with the L. lactis pepN terminator (T1), L. acidophilus slpA terminator (T2), C. difficile slpA terminator (T3), E. coli rrnB terminator T1 loop (T4), B. subtilis Φ29 phage late TD1 terminator (T5), B. subtilis tyrS tRNA terminator (T6), or B. subtilis gyrA terminator (T7).*
