# Supplementary material for: Production of a functional cell wall-anchored minicellulosome by recombinant Clostridium acetobutylicum ATCC 824
Source: Biotechnol Biofuels. 2016 May 23;9:109. doi: 10.1186/s13068-016-0526-x (PMC4877998; doi:10.1186/s13068-016-0526-x)
Supplement: Supplementary file 2 — 10.1186/s13068-016-0526-x SDS-PAGE/western blot of supernatants for sugar release assays. This file contains Figure S2 and Figure S3, comprising the SDS-PAGE/western blot and Coomassie stain analysis of the supernatants used in the sugar release assay shown in Fig. 8. [file 13068_2016_526_MOESM2_ESM.docx]

**Additional File 2: SDS-PAGE/western blot of supernatants for sugar release assays**

Supernatants used in the sugar release assays described in Fig. 8 were subjected to western blot and Coomassie blue staining in order to confirm the presence of the recombinant proteins. Although strain CEL21 produces a cell wall-anchored minicellulosome, the majority of the proteins can be found in the supernatant. Strains were grown to an equivalent OD_600_ to ensure comparability between the levels of produced protein.


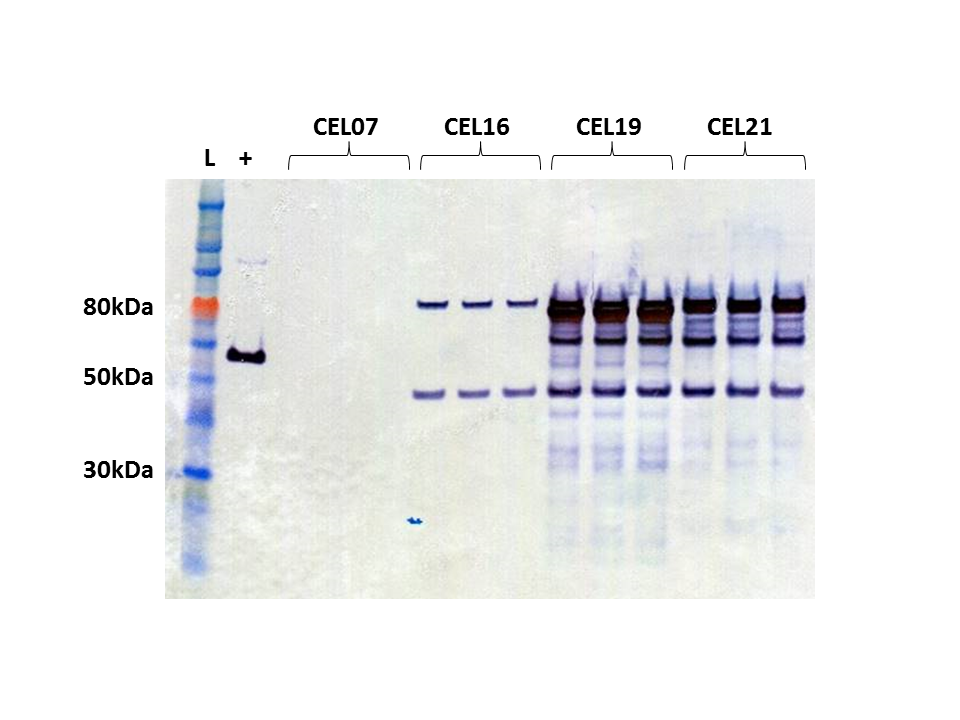


***Figure S2: Western analysis of supernatants from recombinant C. acetobutylicum strains CEL06, CEL16, CEL19 and CEL21 concentrated 10 times by ultrafiltration****. Supernatant was mixed in a 1:1 ratio with 2x SDS sample buffer then subjected to SDS-PAGE followed by western blot using monoclonal ANTI-FLAG M2-peroxidase antibody. L: ColorPlus Prestained Protein Ladder (10-230 kDa); +: Amino-terminal Flag-BAP control protein. CEL06, strain overexpressing the native srtA gene, replicates 1-3; CEL16, producing Xyn10A, Cel9G and Cel48F and overexpressing the native srtA gene, replicates 1-3; CEL19, producing the enzymes, sortase, and unanchored CipC2F3 miniscaffoldin, replicates 1-3; and CEL21, producing the enzymes, sortase and anchored scaffoldin CipC2F3-CA_C0205ss, replicates 1-3.*


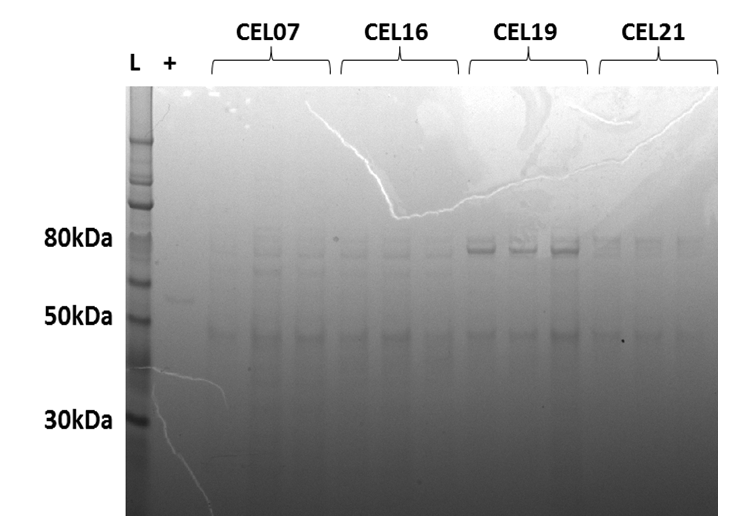


***Figure S3: Coomassie Blue analysis of supernatants from recombinant C. acetobutylicum strains CEL06, CEL16, CEL19 and CEL21 concentrated 10 times by ultrafiltration.*** *Supernatant was mixed in a 1:1 ratio with 2x SDS sample buffer then subjected to SDS-PAGE followed by staining with Coomassie Blue. L: ColorPlus Prestained Protein Ladder (10-230 kDa); +: Amino-terminal Flag-BAP control protein. CEL06, strain overexpressing the native srtA gene, replicates 1-3; CEL16, producing Xyn10A, Cel9G and Cel48F and overexpressing the native srtA gene, replicates 1-3; CEL19, producing the enzymes, sortase, and unanchored CipC2F3 miniscaffoldin, replicates 1-3; and CEL21, producing the enzymes, sortase and anchored scaffoldin CipC2F3-CA_C0205ss, replicates 1-3.*
