# Supplementary material for: Production of a functional cell wall-anchored minicellulosome by recombinant Clostridium acetobutylicum ATCC 824
Source: Biotechnol Biofuels. 2016 May 23;9:109. doi: 10.1186/s13068-016-0526-x (PMC4877998; doi:10.1186/s13068-016-0526-x)
Supplement: Supplementary file 3 — 10.1186/s13068-016-0526-x Fractionation of strains expressing Cel48F-Flag and Xyn10A-Flag. This file contains Figure S4, comprising the western blot analysis of cell fractions of strains expressing Cel48F-Flag and Xyn10A-Flag. [file 13068_2016_526_MOESM3_ESM.docx]

**Additional File 3: Fractionation of hydrolase-expressing strains**

Due to the low amounts of Cel48F present in the supernatants of our recombinant strains, we carried out a cell fractionation on strain CEL12, expressing Cel48F-Flag, in order to confirm that Cel48F was being efficiently secreted. As Xyn10A was observed to be efficiently produced and secreted, strain CEL13, expressing Xyn10A-Flag, was also examined to provide a comparison. Both hydrolases were only detectable in the supernatant fraction, suggesting that Cel48F is wholly secreted at the current levels of expression.

**Supplementary Method: Modified cell fractionation**

Overnight precultures of *C. acetobutylicum* strains were prepared in liquid 2xYTG medium as described in the main text and used to inoculate 20 ml 2xYTG medium to an OD_600_ of 0.05. At exponential phase (OD_600_ 0.7-0.8), cells were harvested for fractionation. A volume equivalent to an OD_600_ of 1 in 1 ml was centrifuged for 1 minute and the supernatant discarded; the pellet was retained and provided the whole cell fraction. Of the remaining medium, a volume equivalent to an OD_600_ of 1 in 10 ml was centrifuged at 5000 g for 10 minutes. Supernatants were retained and concentrated 100-fold via TCA precipitation as described in the main text, providing the supernatant fraction. Cell pellets were washed once in 10 ml pre-reduced lysis buffer, before being resuspended in 10 ml of the same buffer with 3 mg/ml lysozyme. After 3 hours of incubation in the anaerobic cabinet, the entire cell suspension was centrifuged at 10000 g for 30 minutes. 1 ml of the supernatant was removed and concentrated 10-fold via TCA precipitation, providing the cell wall fraction. The pellet was resuspended in 1 ml ice-cold lysis buffer containing Proteinase Inhibitor Cocktail VII (Calbiochem) and lysed by sonication (3 10-second bursts with approximately 1 minute on ice between each). The lysates were centrifuged at full speed at 4°C for 30 minutes; the supernatants were concentrated 10-fold by TCA precipitation, providing the cytoplasmic fraction. The pellet was resuspended in 1 ml lysis buffer containing Proteinase Inhibitor Cocktail VII and 1% Tween 100, and incubated at room temperature for 10 minutes. The suspension was subsequently centrifuged at full speed at 4°C for 10 minutes. The supernatant of this centrifugation was concentrated 10-fold by TCA precipitation, providing the membrane fraction; the pellet contained the insoluble fraction. All fractions were resuspended in 100 µl 2x loading dye + DTT, as described in the main text, and heated to 70°C for 10 minutes.

**
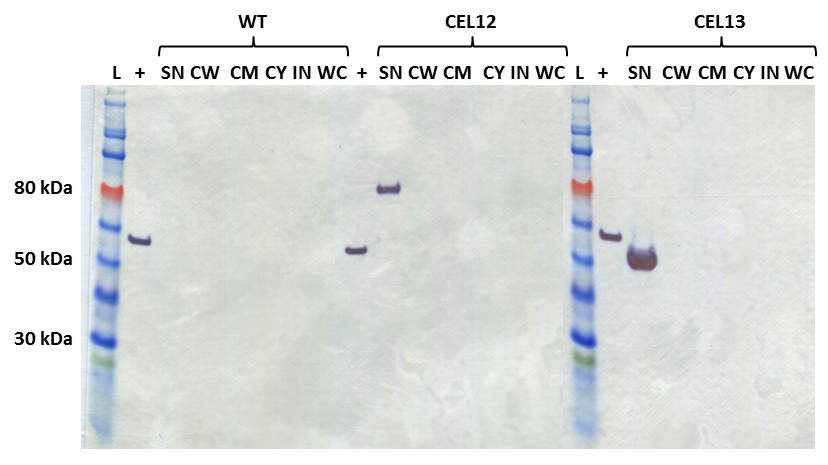
**

***Figure S4: Fractionation of strains expressing the glycoside hydrolases Cel48F and Xyn10A.*** *Strains CEL12, expressing Cel48F-Flag, and CEL13, expressing Xyn10A-Flag, were cultured and fractionated according to the protocol described above. . Proteins were separated on 4-12% Bis-Tris gradient gels; after blotting, cellulosomal components were detected using ANTI-FLAG M2 monoclonal antibody-horseradish peroxidase conjugate. Six fractions were examined: SN, culture supernatant; CW, cell wall; CM, cell membrane; CY, cytoplasm; IN, insoluble; WC, whole cell. L, ColorPlus Prestained Protein Ladder (10–230 kDa); +, Carboxy-terminal FLAG-BAP control protein (50 kDa).*
