# Supplementary material for: Production of a functional cell wall-anchored minicellulosome by recombinant Clostridium acetobutylicum ATCC 824
Source: Biotechnol Biofuels. 2016 May 23;9:109. doi: 10.1186/s13068-016-0526-x (PMC4877998; doi:10.1186/s13068-016-0526-x)
Supplement: Supplementary file 5 — 10.1186/s13068-016-0526-x Plasmids, strains, and oligonucleotides used in the study. This file contains Table S1, Table S2 and Table S3, containing a full list of vectors used in this study, the details of the E. coli and C. acetobutylicum strains used in this study, and a full list of all oligonucleotides, respectively. [file 13068_2016_526_MOESM5_ESM.docx]

**Additional File 5: Plasmids, strains, and oligonucleotides used in the study**

**Table S1: List of vectors constructed during this study**

| Number | Plasmid | Source | Assembly |
| --- | --- | --- | --- |
|  | **Constructs ordered for synthesis** |  |  |
| 1 | pBMH_CBD_X_C1 | Biomatik | Synthesis |
| 2 | pBMH_C2 | Biomatik | Synthesis |
| 3 | pBMH_C3 | Biomatik | Synthesis |
| 4 | pBMH_ScarlessCipC3 | Biomatik | Synthesis |
| 5 | pBMH_BB2thlOid_noRBS | Biomatik | Synthesis |
| 6 | pBMH_BB2fdxOid_noRBS | Biomatik | Synthesis |
| 7 | pBMH_BB2facOid_noRBS | Biomatik | Synthesis |
| 8 | pBMH_CA_C0353ss | Biomatik | Synthesis |
| 9 | pBMH_CA_C0205ss | Biomatik | Synthesis |
| 10 | pBMH_Tcpf_noRBS | Biomatik | Synthesis |
| 11 | pBMH_RBS_CatP | Biomatik | Synthesis |
|  |  |  |  |
|  | **Assembly of scaffoldin constructs** |  |  |
| 12 | pJ202_FLAG_2xStop | This laboratory (unpublished) |  |
| 13 | pJ204_2xStop | This laboratory (unpublished) |  |
| 14 | pJ201_CipC_CBD_X_C1_C2 | This work | pJ201 + 1 + 2 |
| 15 | pJ202_CipC_CBD_X_C1_C2_C3 | This work | pJ202 + 14 + 3 |
| 16 | pJ201_BBCipC3_FLAG | This work | pJ201 + 15 + Annealed oligos |
| 17 | pJ202_ScarlessCipC3_FLAG | This work | pJ202 + 4 + Annealed oligos |
| 18 | pBMH_NCipC_CA_C0353ss | Previous work (unpublished) | 1 + 15 + 8 + Annealed oligos |
| 19 | pBMH_NCipC_CA_C0205ss | Previous work (unpublished) | 1 + 15 + 9 + Annealed oligos |
| 20 | pJ204_NCipC_stop | Previous work (unpublished) | 1 + 15 + 13 + Annealed oligos |
| 21 | pJ201_CipC2F3_CA_C0353ss | This work | pJ201 + 14 + PCR product |
| 22 | pJ201_CipC2F3_CA_C0205ss | This work | pJ201 + 14 + PCR product |
| 23 | pJ201_CipC2F3_stop | This work | pJ201 + 14 + PCR product |
| 24 | pJ202_RBS_SaSrtA_nostop | This work | pJ202 + thlRBS + PCR product |
| 25 | pJ204_RBS_SaSrtA_stop | This work | 13 + 24 |
| 26 | pJ204_CipC2F3_CA_C0205ss_SaSrtA | This work | pJ201 + 19 + PCR product |
|  |  |  |  |
|  | **Analysis of terminator efficacies** |  |  |
| 27 | pJ201_lacIQ | Previous work [29] |  |
| 28 | pJ201_CipA2_FLAG | Previous work [29] |  |
| 29 | pJ201_lacIQ_TpepN | This work | 27 + annealed oligos |
| 30 | pJ201_lacIQ_TslpA_LA | This work | 27 + annealed oligos |
| 31 | pJ201_lacIQ_TslpA_CD | This work | 27 + annealed oligos |
| 32 | pJ201_lacIQ_EcoT1 | This work | 27 + annealed oligos |
| 33 | pJ201_lacIQ_phiTD1 | This work | 27 + annealed oligos |
| 34 | pJ201_lacIQ_TtyrS | This work | 27 + annealed oligos |
| 35 | pJ201_lacIQ_TgyrA | This work | 27 + annealed oligos |
| 36 | pJ201_lacIQ_TpepN_CipA2_FLAG | This work | 30 + 29 |
| 37 | pJ201_lacIQ_TslpA_LA_CipA2_FLAG | This work | 31 + 29 |
| 38 | pJ201_lacIQ_TslpA_CD_CipA2_FLAG | This work | 32 + 29 |
| 39 | pJ201_lacIQ_EcoT1_CipA2_FLAG | This work | 33 + 29 |
| 40 | pJ201_lacIQ_phiTD1_CipA2_FLAG | This work | 34 + 29 |
| 41 | pJ201_lacIQ_TtyrS_CipA2_FLAG | This work | 35 + 29 |
| 42 | pJ201_lacIQ_TgyrA_CipA2_FLAG | This work | 36 + 29 |
|  |  |  |  |
|  | **Assembly of enzyme constructs** |  |  |
| 43 | pJ201_Cel9G_wt | This laboratory (unpublished) |  |
| 44 | pJ202_Cel48F_wt | This laboratory (unpublished) |  |
| 45 | pJ202_Xyn10A_wt | This laboratory (unpublished) |  |
| 46 | pJ202_Cel9G_FLAG | This work | pJ202 + 43 + annealed oligos |
| 47 | pJ201_Cel48F_FLAG | This work | pJ201 + 44 + annealed oligos |
| 48 | pJ202_Xyn10A_FLAG | This work | 12 + 45 |
| 49 | pJ201_lacIQ_CatP | This work | 27 + 11 |
| 50 | pJ201_lacIQ_BB2thlOid_CatP | This work | 49 + 5 |
| 51 | pJ201_lacIQ_BB2fdxOid_CatP | This work | 49 + 6 |
| 52 | pJ201_lacIQ_BB2facOid_CatP | This work | 49 + 7 |
| 53 | pJ201_lacIQ_BB2facOid_Xyn10A_FLAG | This work | 52 + 48 |
| 54 | pJ201_lacIQ_BB2thlOid_Xyn10A_FLAG | This work | 50 + 48 |
| 55 | pJ201_lacIQ_BB2thlOid_Xyn10A_FLAG_TtyrS | This work | 34 + 54 |
| 56 | pJ201_lacIQ_BB2fdxOid_Cel9G_FLAG | This work | 51 + 46 |
| 57 | pJ201_lacIQ_BB2facOid_Cel48F_FLAG | This work | 52 + 47 |
| 58 | pJ201_lacIQ_BB2fdxOid_Cel9G_FLAG_EcoT1 | This work | 32 + 56 |
| 59 | pJ201_lacIQ_BB2fdxOid_Cel9G_FLAG_EcoT1_BB2facOid_Cel48F_FLAG | This work | 57 + 58 |
|  |  |  |  |
|  | **Integration vectors** |  |  |
| 60 | pMTL-JH16 | This laboratory [42] |  |
| 61 | pMTL-JH16_TpepN_CipA2_FLAG | This work | 60 + 36 |
| 62 | pMTL-JH16_TslpA_LA_CipA2_FLAG | This work | 60 + 37 |
| 63 | pMTL-JH16_TslpA_CD_CipA2_FLAG | This work | 60 + 38 |
| 64 | pMTL-JH16_EcoT1_CipA2_FLAG | This work | 60 + 39 |
| 65 | pMTL-JH16_phiTD1_CipA2_FLAG | This work | 60 + 40 |
| 66 | pMTL-JH16_TtyrS_CipA2_FLAG | This work | 60 + 41 |
| 67 | pMTL-JH16_TgyrA_CipA2_FLAG | This work | 60 + 42 |
| 68 | pMTL-JH16_lacIQ | Previous work [29] |  |
| 69 | pMTL-JH16_BBCipC3_FLAG | This work | 60 + 16 |
| 70 | pMTL-JH16_ScarlessCipC3_FLAG | This work | 60 + 17 |
| 71 | pMTL-JH16_CipC2F3_CA_C0353ss | This work | 60 + 21 |
| 72 | pMTL-JH16_CipC2F3_CA+C0205ss | This work | 60 + 22 |
| 73 | pMTL-JH16_CipC2F3_stop | This work | 60 + 23 |
| 74 | pMTL-JH16_CipC2F3_CA_C0205ss_SaSrtA | This work | 60 + 26 |
| 75 | pMTL-JH14 | This laboratory [42] |  |
| 76 | pMTL-JH14_lacIQ | This work | 75 + 68 |
| 77 | pMTL82254_facOid_CatP | This laboratory (unpublished) |  |
| 78 | pMTL-JH14_lacIQ_facOid | This work | 76 + 77 |
| 79 | pMTL-JH14_lacIQ_facOid_Cel9G-FLAG | This work | 78 + 46 |
| 80 | pMTL-JH14_lacIQ_facOid_Cel48F_FLAG | This work | 78 + 47 |
| 81 | pMTL-JH14_lacIQ_BB2facOid_Xyn10A_FLAG | This work | 78 + 53 |
| 82 | pMTL-JH12 | This laboratory [42] |  |
| 83 | pMTL-JH12_lacIQ | This work | 82 + 68 |
| 84 | pMTL-JH12_lacIQ_GF | This work | 83 + 59 |
| 85 | pBW1 | This work | 76 + PCR fragment |
| 86 | pBW1_BB2facOid_Xyn10A-FLAG_TtyrS | This work | 85 + 55 |
|  |  |  |  |
|  | **Sortase expression plasmids** |  |  |
| 87 | pJ201_lacIQ_Tcpf_CatP | This work | 49 + 10 |
| 88 | pMTL82151 | This laboratory | Synthesis |
| 89 | pMTL82151_Tcpf_CatP | This work | 88 + 87 |
| 90 | pMTL82151_Tcpf_CaSrtA | This work | 89 + PCR fragment |
| 91 | pMTL82151_Tcpf_LmSrtA | This work | 89 + PCR fragment |
| 92 | pMTL82151_Tcpf_BcSrtA | This work | 89 + PCR fragment |

This table contains a full list of plasmids used during the study. The source of each plasmid and the assembly strategy have been indicated; numbers in the ‘Assembly’ section refer to the relevant plasmids used for assembly. In the ‘Assembly’ section, plasmids labelled ‘synthesis’ were synthesised by the indicated company, while fragments described as ‘PCR product’ were generated by PCR, and those described as ‘annealed oligos’ were generated by the annealing of two complementary oligonucleotides.

**Table S2: List of bacterial strains used during the study**

| Strain | Relevant genotype | Details |
| --- | --- | --- |
| *E. coli* |  |  |
| Top10 |  | Cloning strain, ordered from ThermoFisher |
| pAN-2 | Contains pAN-2 plasmid [84] | Methylation host for transformation into *C. acetobutylicum* |
| *C. acetobutylicum* |  |  |
| ATCC 824 |  | Deletion of genes from CA_P0066 to CA_P0071 [71] |
| *pyrE*^-^ |  | Disruption of *pyrE* gene by integration of pMTL-JH12 |
| CEL01 | thl:BBCipC3_FLAG | Integration of a gene encoding BBCipC3_FLAG (BioBrick-2 assembled CipC3-FLAG) at the *thl* locus |
| CEL02 | thl:ScarlessCipC3_FLAG | Integration of a gene encoding ScarlessCipC3_FLAG (single fragment CipC3-FLAG) at the *thl* locus |
| CEL03 | thl:CipC2F3 | Integration of a gene encoding CipC2F3 (a CipC3 variant with FLAG-tag located between the second and third cohesins) into the *thl* locus |
| CEL04 | thl:CipC2F3-CA_C0353ss | Integration of a gene encoding CipC2F3-CA_C0353ss (CipC2F3 fused to the cell wall sorting signal of the cyclic AMP phosphorylase CA_C0353) into the *thl* locus |
| CEL05 | thl:CipC2F3-CA_C0205ss | Integration of a gene encoding CipC2F3-CA_0205ss (CipC2F3 fused to the cell wall sorting signal of the Icc-family phosphohydrolase CA_C0205) into the *thl* locus |
| CEL06 | thl:CipC2F3-CA_C0205ss_SaSrtA | Integration of an operon consisting of CipC2F3-CA_C0205ss and *S. aureus srtA* into the *thl* locus |
| CEL07 | pMTL82151:CaSrtA | Introduction of the pMTL82151_Tcpf_CaSrtA vector, leading to expression of *C. acetobutylicum* *srtA* (CA_C0204) from the P_Tcpf_ promoter (BB2-format *C. perfringens* thiolase promoter with *C. acetobutylicum* *thl* RBS) |
| CEL08 | thl:CipC2F3-CA_C0205ss pMTL82151:CaSrtA | Introduction of the pMTL82151_Tcpf_CaSrtA vector into strain CEL05 |
| CEL09 | thl:CipC2F3-CA_C0205ss pMTL82151:LmSrtA | Introduction into strain CEL05 of the pMTL82151_Tcpf_LmSrtA vector, containing the *L. monocytogenes* *srtA* gene under the control of the P_Tcpf_ promoter |
| CEL10 | thl:CipC2F3-CA_C0205ss pMTL82151:BcSrtA | Introduction into strain CEL05 of the pMTL82151_Tcpf_BcSrtA vector, containing the *B. cereus srtA* gene under the control of the P_Tcpf_ promoter |
| CEL11 | pyrE:Cel9G_FLAG | Integration of facOid_Cel9G_FLAG (a gene encoding Cel9G-FLAG under the transcriptional control of P_facOid_, the *C. pasteurianum* ferredoxin promoter with introduced ideal *lac* operator) at the *pyrE* locus |
| CEL12 | pyrE:Cel48F_FLAG | Integration of facOid_Cel9G_FLAG (a gene encoding Cel48F-FLAG under the transcriptional control of P_facOid_) at the *pyrE* locus |
| CEL13 | pyrE:Xyn10A_FLAG | Integration of BB2facOid_Xyn10A_Flag (a gene encoding Xyn10A-FLAG under the transcriptional control of P_BB2facOid_, the BB2-format *C. pasteurianum* ferredoxin promoter with introduced ideal *lac* operator and *C. acetobutylicum* *thl* RBS) at the *pyrE* locus |
| CEL14 | pyrE^-^:GF | Truncation of *pyrE* and the integration of BB2fdxOid_Cel9G_Flag_EcoT1_BB2facOid_Cel48F_Flag (cassette consisting of a gene encoding Cel9G-FLAG under the control of P_BB2fdxOid_, the BB2-format *C. sporogenes fdx* promoter with introduced ideal *lac* operator, and *C. acetobutylicum thl* RBS; the EcoT1 terminator; and a gene encoding Cel48F-FLAG under the control of P_BB2facOid_) at the *pyrE* locus |
| CEL15 | pyrE:XGF | Integration of the XGF gene cassette at the pyrE locus. The XGF gene cassette consists of a gene encoding Xyn10-FLAG under the control of P_BB2thlOid_, the BB2-format *thl* promoter with introduced ideal *lac* operator; the TtyrS terminator; a gene encoding Cel9G-FLAG under the control of P_BB2fdxOid_; the EcoT1 terminator; and a gene encoding Cel48F-FLAG under the control of P_BB2facOid_. |
| CEL16 | pyrE:XGF pMTL82151:CaSrtA | Introduction of the pMTL82151_Tcpf_CaSrtA vector into strain CEL15 |
| CEL17 | pyrE:XGF thl:CipC3 | Integration of a gene encoding BBCipC3-FLAG at the *thl* locus and the XGF gene cassette at the *pyrE* locus |
| CEL18 | pyrE:XGF thl:CipC2F3 | Integration of a gene encoding CipC2F3 at the *thl* locus and the XGF gene cassette at the *pyrE* locus |
| CEL19 | pyrE:XGF thl:CipC2F3 pMTL82151:CaSrtA | Introduction of the pMTL82151_Tcpf_CaSrtA vector into strain CEL18 |
| CEL20 | pyrE:XGF thl:CipC2F3-CA_C0205ss | Integration of a gene encoding CipC2F3-CA_C0205ss at the *thl* locus and the XGF gene cassette at the *pyrE* locus |
| CEL21 | pyrE:XGF thl:CipC2F3-CA_C0205ss pMTL82151:CaSrtA | Introduction of the pMTL82151_Tcpf_CaSrtA vector into strain CEL20 |
| A2 | thl:CipA2 | Integration of a gene encoding CipA2-Flag at the *thl* locus [29] |
| T1 | thl:TpepN_CipA2 | Integration of TpepN_CipA2 (gene encoding CipA2 downstream of the *L. lactis pepN* terminator) at the *thl* locus |
| T2 | thl:TslpA_LA_CipA2 | Integration of TslpA_LA_CipA2 (gene encoding CipA2 downstream of the *L. acidophilus slpA* terminator) at the *thl* locus |
| T3 | thl:TslpA_CD_CipA2 | Integration of TslpA_CD_CipA2 (gene encoding CipA2 downstream of the *C. difficile slpA* terminator) at the *thl* locus |
| T4 | thl:EcoT1_CipA2 | Integration of EcoT1_CipA2 (gene encoding CipA2 downstream of the *E. coli rrnB* T1 terminator) at the *thl* locus |
| T5 | thl:phiTD1_CipA2 | Integration of phiTD1_CipA2 (gene encoding CipA2 downstream of the *B. subtilis* Φ29 phage late TD1 terminator) at the *thl* locus |
| T6 | thl:TtyrS_CipA2 | Integration of TtyrS_CipA2 (gene encoding CipA2 downstream of the *B. subtilis tyrS* tRNA terminator) at the *thl* locus |
| T7 | thl:TgyrA_CipA2 | Integration of TgyrA_CipA2 (gene encoding CipA2 downstream of the *B. subtilis gyrA* terminator) at the *thl* locus |

This table contains the details of the *E. coli* and *C. acetobutylicum* strains used during this study, and provides details of the modifications made to the recombinant *C. acetobutylicum* strains.

**Table S3: Oligonucleotides used during this study**

| Oligonucleotide | Sequence |
| --- | --- |
| Flag_fw | GAATTCGCGGCCGCACTAGTGATTACAAGGATGACGACGATAAGTAATGAGCTAGCGCGGCCGCCTGCAG |
| Flag_rev | CTGCAGGCGGCCGCGCTAGCTCATTACTTATCGTCGTCATCCTTGTAATCACTAGTGCGGCCGCGAATTC |
| TpepN_fd | gcGAATTCGCGGCCGCACTAGTtaatttataaataaaaatcaccttttagaggtggtttttttatttataaattaGCTAGCGCGGCCGCCTGCAGcg |
| TpepN_rev | cgCTGCAGGCGGCCGCGCTAGCTAATTTATAAATAAAAAAACCACCTCTAAAAGGTGATTTTTATTTATAAATTAACTAGTGCGGCCGCGAATTCgc |
| TslpA_LA_fd | gcGAATTCGCGGCCGCACTAGTtgaaaaaggcagagcgaaagctctgtcttttttGCTAGCGCGGCCGCCTGCAGcg |
| TslpA_LA_rev | cgCTGCAGGCGGCCGCGCTAGCAAAAAAGACAGAGCTTTCGCTCTGCCTTTTTCAACTAGTGCGGCCGCGAATTCgc |
| TslpA_CD_fd | gcGAATTCGCGGCCGCACTAGTaaatataaaaagacttctcagatgagaagtcttttttgtgaaaGCTAGCGCGGCCGCCTGCAGcg |
| TslpA_CD_rev | cgCTGCAGGCGGCCGCGCTAGCTTTCACAAAAAAGACTTCTCATCTGAGAAGTCTTTTTATATTTACTAGTGCGGCCGCGAATTCgc |
| EcoT1_fd | gcGAATTCGCGGCCGCACTAGTccaggcatcaaataaaacgaaaggctcagtcgaaagactgggcctttcgttttatct  gttgtttgtcggtgaacgctctcGCTAGCGCGGCCGCCTGCAGcg |
| EcoT1_rev | cgCTGCAGGCGGCCGCGCTAGCGAGAGCGTTCACCGACAAACAACAGATAAAACGAAAGGCCCAGTCTTTCGACTGAGCCTTTCGTTTTATTTGATGCCTGGACTAGTGCGGCCGCGAATTCgc |
| phiTD1_fd | gcGAATTCGCGGCCGCACTAGTaacaatcaaaagaaaagcctatcgtctgaggaacggtaggctcttttgtagcatata  gttgGCTAGCGCGGCCGCCTGCAGcg |
| phiTD1_rev | cgCTGCAGGCGGCCGCGCTAGCCAACTATATGCTACAAAAGAGCCTACCGTTCCTCAGACGATAGGCTTTTCTTTTGATTGTTACTAGTGCGGCCGCGAATTCgc |
| TtyrS_fd | gcGAATTCGCGGCCGCACTAGTataatcaatcgtcccttcgtgtaaacgaaggggcgttttttatttGCTAGCGCGGCCGCCTGCAGcg |
| TtyrS_rev | cgCTGCAGGCGGCCGCGCTAGCAAATAAAAAACGCCCCTTCGTTTACACGAAGGGACGATTGATTATACTAGTGCGGCCGCGAATTCgc |
| TgyrA_fd | gcGAATTCGCGGCCGCACTAGTaagaagaagtgtgaaaaagcgcagctgaaatagctgcgcttttttgtgtcataaGCTAGCGCGGCCGCCTGCAGcg |
| TgyrA_rev | cgCTGCAGGCGGCCGCGCTAGCTTATGACACAAAAAAGCGCAGCTATTTCAGCTGCGCTTTTTCACACTTCTTCTTACTAGTGCGGCCGCGAATTCgc |
| 9G_arm_fd | aattGCTAGCGTGTAGTAGCCTGTGAAATAAGTAAGG |
| 9G_arm_rev | aattGGCGCGCCATATACAGATACTTTGGATGGCGTACAGC |
| C2FC3FD | ttaaACTAGTGATTACAAGGATGACGACGATAAGCAACCTACAAAGGAACTTAAAGTAGC |
| C2FC3_BMHrev | GAAACAGCTATGACCATGATTACG |
| C2FC3_204rev | CTTAGGTACGAACTCGATTGACG |
| SrtA_start_fd | ttaaCATATGAAAAAATGGACAAATCGATTAATGACAATCG |
| SrtA_nostop_rev | ttaaGCTAGCTTTGACTTCTGTAGCTACAAAGATTTTACG |
| CaSrtA_fd | aaaCATATGAAAAAGTTAAACATTATAGCAGCTACACTTATATCATCAGG |
| CaSrtA_rev | aaaGCTAGCTTAAATTCTTTTAGCTTTTATAATAAGTCTATGAGTAGCTATTCTTATAGG |
| Bcer_fw | cgcatAtgaataagcaaagaatttatagtatagtagc |
| Bcer_rev | agcgctagcttacttcttcgccttcgttcttact |
| Gb_Lm_Fw | agtgtatcaaaatttaggaggttagttCatatgttaaagaaaacaattgcaataataattttaatcatcgggct |
| Gb_Lm_Rev | atacttttccttcatcaaaattcccccaaaatgctagcttatttactagggaaatatttattctctaattct |

This table contains a list of the oligonucleotides used during this study. Bases in lower case indicate those that are included solely for the purpose of facilitating subsequent restriction digests, and are thus not included in the final constructs. Oligonucleotides were ordered from Eurofins MWG Operon.
