# Supplementary material for: Production of a functional cell wall-anchored minicellulosome by recombinant Clostridium acetobutylicum ATCC 824
Source: Biotechnol Biofuels. 2016 May 23;9:109. doi: 10.1186/s13068-016-0526-x (PMC4877998; doi:10.1186/s13068-016-0526-x)
Supplement: Supplementary file 6 — 10.1186/s13068-016-0526-x Sequences of the proteins and genetic components used in the study. This file contains the amino acid sequences of the proteins used in this study, as well as the nucleotide sequences of the promoters and terminators used in the construction of the XGF gene cassette and sortase expression vectors. [file 13068_2016_526_MOESM6_ESM.docx]

**Additional File 6: Sequences of the proteins, promoters and terminators used in the study**

**Mini-CipC scaffoldins** (based on full-length CipC sequence [GenBank: AAC28899.2])

MRKKSLAFLLALTMLVTLLGAQLTAFAAGTGVVSVQFNNGSSPASSNSIYARFKVTNTSGSPINLADLKLRYYYTQDADKPLTFWCDHAGYMSGSNYIDATSKVTGSFKAVSPAVTNADHYLEVALNSDAGSLPAGGSIEIQTRFARNDWSNFDQSNDWSYTAAGSYMDWQKISAFVGGTLAYGSTPDGGNPPPQDPTINPTSISAKAGSFADTKITLTPNGNTFNGISELQSSQYTKGTNEVTLLASYLNTLPENTTKTLTFDFGVGTKNPKLTITVLPKDIPGDSLKVTVGTANGKPGDTVTVPVTFADVAKMKNVGTCNFYLGYDASLLEVVSVDAGPIVKNAAVNFSSSASNGTISFLFLDNTITDELITADGVFANIKFKLKSVTAKTTTPVTFKDGGAFGDGTMSKIASVTKTNGSVTIDPGTQPTKELKVAVGTANGKPGDTVTVPVTFADVVNVGNVGTCNFYLGYDASLLEVVSVDAGPIVKNAAVNFSSSASNGTISFLFLDNTITDELITSDGVFANIKFKLKSVATKTTTPVTFKDGGAFGDGTMAKIATVTKTNGSVTIDPGTDYKDDDDKQPTKELKVAVGTANGKPGDTVTVPVTFADVASAGNVGTCNFYLAYDASLLEVVSVDAGPIVKNAAVNFSSSASNGSISFLFLDNTITDELITADGVFANIKFKLKSVAAKTTTPVTFKDGGAFGDGTMTKIATVTKTNGSVTIDPASDYKDDDDK

Amino acid sequence of mini-CipC3 variants (BBCipC3-FLAG, ScarlessCipC3-FLAG, CipC2F3) assembled from BioBrick-2 format parts. Residues with grey highlight make up the signal peptide, blue residues represent the inter-domain flexible linker regions, green highlights represent residues replaced by alanine-serine scars in BBCipC3 and CipC2F3 as a result of BB2 assembly, and orange residues represent the location of the FLAG epitope tag in CipC2F3, while yellow residues represent the FLAG epitope tag in BBCipC3-FLAG and ScarlessCipC3-FLAG.

**Sortase signal sequences**

CA_C0353ss (Derived from *C. acetobutylicum* ATCC 824 2,3-cyclic-nucleotide 2’ phosphodiesterase [NCBI:NP_346993.1])

LPKTGSMIDSTVLLIIGTLLLLLGLAFIIWNKFKNKQKSVQ

CA_C0205ss (Derived from *C. acetobutylicum* ATCC 824 Icc-family phosphohydrolase [NCBI:NP_346847.1])

LPKTGEFFDATMLLSIALICLASGAILIFVNKKKSSPTK

Sortase signal sequences used in the study. The sequences above were attached to the C-terminus of CipC2F3 to generate CipC2F3-CA_C0353ss and CipC2F3-CA_C0205ss respectively. Blue highlight represents the LPXTG motif, whereas green highlight represents the positively charged C-terminal region.

**Sortases**

Sa-SrtA (*S. aureus* SrtA [NCBI: WP_000759361.1])

MKKWTNRLMTIAGVVLILVAAYLFAKPHIDNYLHDKDKDEKIEQYDKNVKEQASKDKKQQAKPQIPKDKSKVAGYIEIPDADIKEPVYPGPATPEQLNRGVSFAEENESLDDQNISIAGHTFIDRPNYQFTNLKAAKKGSMVYFKVGNETRKYKMTSIRDVKPTDVGVLDEQKGKDKQLTLITCDDYNEKTGVWEKRKIFVATEVK

Ca-SrtA (*C. acetobutylicum* ATCC 824 sortase [NCBI: NP_346846.1])

MKKLNIIAATLISSGVILIGFTLGAKYWTYHKQNSMINAYEKKIKNIKHSNNKKVKAAEIKDTIKDGTIGILKIPKIDLKVAIGEGTDLKTLKYAVGHFRNTSMPGQNGNFCLAGHRSYTFGEYFNRLGEIGSGDEIDVETVNGTFKYKVYSTKVVLPSEVHVLDQTKDPTMTLVTCTPIRIATHRLIIKAKRI

Lm-SrtA (*L. monocytogenes* EGD Sortase A, LPXTG specific [GenBank: CDG44797.1])

MLKKTIAIIILIIGLLLIFSPFIKNGIVKYMSGHETIEQYKASDIKKNNEKDATFDFESVQLPSMTSVIKGAANYDKDAVVGSIAVPSVDVNLLVFKGTNTANLLAGATTMRSDQVMGKGNYPLAGHHMRDESMLFGPIMKVKKGDKIYLTDLENLYEYTVTETKTIDETEVSVIDNTKDARITLITCDKPTETTKRFVAVGELEKTEKLTKELENKYFPSK

Bc-SrtA (*B. cereus* ATCC 10987 LPXTG-site transpeptidase protein [GenBank: AAS39688.1])

MNKQRIYSIVAILLFVVGGVLIGKPFYDGYQAEKKQTENVQAVQKMDYEKHETEFVDASKINQPDLAEVANASLDKKQVIGRISIPSVSLELPVLKSSTEKNLLSGAATVKENQVMGKGNYALAGHNMSKKGVLFSDIASLKKGDKIYLYDNENEYEYAVTGVSEVTPDKWEVVEDHGKDEITLITCVSVKDNSKRYVVAGDLVGTKAKK

Amino acid sequences of the sortase enzymes used in the study.

**Glycoside hydrolases**

Cel9G-FLAG (derived from Cel9G [GenBank: ACL75110.1])

MLKTKRKLTKAIGVALSISILSSLVSFIPQTNTYAAGTYNYGEALQKSIMFYEFQRSGDLPADKRDNWRDDSGMKDGSDVGVDLTGGWYDAGDHVKFNLPMSYTSAMLAWSLYEDKDAYDKSGQTKYIMDGIKWANDYFIKCNPTPGVYYYQVGDGGKDHSWWGPAEVMQMERPSFKVDASKPGSAVCASTAASLASAAVVFKSSDPTYAEKCISHAKNLFDMADKAKSDAGYTAASGYYSSSSFYDDLSWAAVWLYLATNDSTYLDKAESYVPNWGKEQQTDIIAYKWGQCWDDVHYGAELLLAKLTNKQLYKDSIEMNLDFWTTGVNGTRVSYTPKGLAWLFQWGSLRHATTQAFLAGVYAEWEGCTPSKVSVYKDFLKSQIDYALGSTGRSFVVGYGVNPPQHPHHRTAHGSWTDQMTSPTYHRHTIYGALVGGPDNADGYTDEINNYVNNEIACDYNAGFTGALAKMYKHSGGDPIPNFKAIEKITNDEVIIKAGLNSTGPNYTEIKAVVYNQTGWPARVTDKISFKYFMDLSEIVAAGIDPLSLVTSSNYSEGKNTKVSGVLPWDVSNNVYYVNVDLTGENIYPGGQSACRREVQFRIAAPQGTTYWNPKNDFSYDGLPTTSTVNTVTNIPVYDNGVKVFGNEPAGGSENPDPEILYGDVNSDKNVDALDFAALKKYLLGGTSSIDVKAADTYKDGNIDAIDMATLKKYLLGTITQLPQGASDYKDDDDK

Cel48F-FLAG (Derived from Cel48F [GenBank: ACL75108.1])

MSKNFKRVGAVAVAAAMSLSIMATTSINAASSPANKVYQDRFESMYSKIKDPANGYFSEQGIPYHSIETLMVEAPDYGHVTTSEAMSYYMWLEAMHGRFSGDFTGFDKSWSVTEQYLIPTEKDQPNTSMSRYDANKPATYAPEFQDPSKYPSPLDTSQPVGRDPINSQLTSAYGTSMLYGMHWILDVDNWYGFGARADGTSKPSYINTFQRGEQESTWETIPQPCWDEHKFGGQYGFLDLFTKDTGTPAKQFKYTNAPDADARAVQATYWADQWAKEQGKSVSTSVGKATKMGDYLRYSFFDKYFRKIGQPSQAGTGYDAAHYLLSWYYAWGGGIDSTWSWIIGSSHNHFGYQNPFAAWVLSTDANFKPKSSNGASDWAKSLDRQLEFYQWLQSAEGAIAGGATNSWNGRYEAVPSGTSTFYGMGYVENPVYADPGSNTWFGMQVWSMQRVAELYYKTGDARAKKLLDKWAKWINGEIKFNADGTFQIPSTIDWEGQPDTWNPTQGYTGNANLHVKVVNYGTDLGCASSLANTLTYYAAKSGDETSRQNAQKLLDAMWNNYSDSKGISTVEQRGDYHRFLDQEVFVPAGWTGKMPNGDVIKSGVKFIDIRSKYKQDPEWQTMVAALQAGQVPTQRLHRFWAQSEFAVANGVYAILFPDQGPEKLLGDVNGDETVDAIDLAILKKYLLNSSTTINTANADMNSDNAIDAIDYALLKKALLSIQASDYKDDDDK

Xyn10A-FLAG (Derived from Xyn10A [GenBank: ACL75297.1])

MRKNSFKSLAVALSVLLTALMVCSSMVSAATPTGKRLKDVQSRVLVGTEFSSGFTNMDSTFFNTATPEFNLVTAENCMKWDALEPSQNSFNWNEADKLMNWAKTNNYKVHGHTFVWHNQAPGWIQNLSASAMESAMNNHIDKVMGRYKGQIPIWDVANEVFEENGSYRNSFWYRTMGKSFIEKAFIRARAADPSAKLVYNDYNLEYTGPKSNAAYEMLKDFKSRGIPVDGIGFQMHLDIQYAIDYNDFAKNMQRFADLGLEIYITEMDVRVSSNTNSTELQTQASYYKNIIEKCMAQPAVKAIQFWGFTDKYSWVPGTFSGRDNALLFDKNYNPKPAYYAVQAALATSPTPTVIYGDLDGSGSVDALDYSLMKQYLLGSITKFPSENGLVAADVNASGTVDALDFAVMKQYLIGLITKFPAQVASDYKDDDDK

Amino acid sequences of the glycoside hydrolase enzymes used in the study, Grey highlight represents the predicted signal peptide, green highlight represents the alanine-serine scar produced by BB2 assembly, and yellow highlight represents the FLAG epitope tag.

**Promoter sequences**

P_BB2_*_thl_*_Oid_ (derived from the *C. acetobutylicum* ATCC 824 *thl* gene promoter [GenBank: AE001437.

TTTTTAACAAAATATATTGATAAAAATAATAATAGTGGGTATAATTAATGTGGAATTGTGAGCGCTCACAATTGGTGTGTTGTTAGAGAAAACGTATAAATTAGGGATAAACTATGGAACTTATGAAATAGATTGAAATGGTTTATCTGTGCTAGTGTATCAAAATTTAGGAGGTTAGTTCAT**ATG**

P_BB2_*_fdx_*_Oid_ (derived from the *C. sporogenes* DSM 795 *fdx* gene promoter [GenBank: CP011663.1, 86273-86444])

GTGTAGTAGCCTGTGAAATAAGTAAGGAAAAAAAAGAAGTAAGTGTTATATATGATGATTATTTTGTAGATGTAGATAGGATAATAGAATCCATAGAAAATATAGGTTATACAGTTATATAAAAATTACTTTAAAAATTAATAAAAACATGGTAAAATATAAATCGTATAAATGTGGAATTGTGAGCGCTCACAATTGGTGTGCTAGTGTATCAAAATTTAGGAGGTTAGTTCAT**ATG**

­P_BB2_*_fac_*_Oid_ (derived from the *C. pasteurianum* *fdx* gene promoter [GenBank: M11214.1, 5-131])

GAGATAGTATATGATGCATATTCTTTAAATATAGATAAAGTTATAGAAGCAATAGAAGATTTAGGATTTACTGTAATATAAATTACACTTTTAAAAAGTTTAAAAACATGATACAATAAGTTATGGTTGGTGTGGAATTGTGAGCGCTCACAATTGGTGTCCAAGCTAGTGTATCAAAATTTAGGAGGTTAGTTCAT**ATG**

P*_fac_*_Oid_ (derived from the *C. pasteurianum* *fdx* gene promoter [GenBank: M11214.1, 5-131])

GAGATAGTATATGATGCATATTCTTTAAATATAGATAAAGTTATAGAAGCAATAGAAGATTTAGGATTTACTGTAATATAAATTACACTTTTAAAAAGTTTAAAAACATGATACAATAAGTTATGGTTGGTGTGGAATTGTGAGCGCTCACAATTGGTGTCCAACTTATGATTAAAATTTTAAGGAGGTGTATTTCAT**ATG**

P_T_*_cpf_* (derived from the *C. perfringens* str. 13 *thl* gene promoter [GenBank: BA000016.3, 2516505-2516740)

GACTTTGTTAAAAAAGTTTAATAAATATAATTTGAATAAATGGTATAAATAGACAGATATTTAGAATATTATAGAAATTTTAATAAAAGACTTCTATAATAAAGCTAAATTATCTGTCTTTTTTTTCGAAAAGAGAAAAAATAATAAAAAAGATTGTTTAAAATTTAACAAAAAATATTGAAAGCTAGTAAATTTATGGTTATAATTAAATCAGTTAGAAAAAATATAGATTTTTGGCTAGTGTATCAAAATTTAGGAGGTTAGTTCAT**ATG**

Nucleotide sequences of the promoters used in this study. All promoters (except P*_fac_*_Oid_) were constructed in BB2 format, with the BB2 suffix located 18bp upstream of the predicted ribosome binding site. BB2 cloning was then used to add a sequence encoding the *C. acetobutylicum* ATCC 824 *thl* RBS and 12bp upstream sequence. Yellow highlights represent the introduced ‘ideal’ *lac* operator sequence, green highlights represent the introduced GCTAGT BB2 scar, and bold/underlined sequences represent the start codon.

**Terminator sequences**

TpepN (*L. lactis pepN* terminator)

taatttataaataaaaatcaccttttagaggtggtttttttatttataaatta

TslpA_LA (*L. acidophilus slpA* terminator)

tgaaaaaggcagagcgaaagctctgtctttttt

TslpA_CD (*C. difficile slpA* terminator)

aaatataaaaagacttctcagatgagaagtcttttttgtgaaa

EcoT1 (*E. coli* *rrnB* terminator T1 loop)

Ccaggcatcaaataaaacgaaaggctcagtcgaaagactgggcctttcgttttatctgttgtttgtcggtgaacgctctc

phiTD1 (*B. subtilis* phage Φ29 late TD1 terminator)

aacaatcaaaagaaaagcctatcgtctgaggaacggtaggctcttttgtagcatatagttg

TtyrS (*B. subtilis* *tyrS* tRNA terminator)

ataatcaatcgtcccttcgtgtaaacgaaggggcgttttttattt

TgyrA (*B. subtilis gyrA* terminator)

aagaagaagtgtgaaaaagcgcagctgaaatagctgcgcttttttgtgtcataa

Nucleotide sequences of the terminators used in this study.
